# High-Throughput RNA Sequencing of Pseudomonas-Infected Arabidopsis Reveals Hidden Transcriptome Complexity and Novel Splice Variants

**DOI:** 10.1371/journal.pone.0074183

**Published:** 2013-10-01

**Authors:** Brian E. Howard, Qiwen Hu, Ahmet Can Babaoglu, Manan Chandra, Monica Borghi, Xiaoping Tan, Luyan He, Heike Winter-Sederoff, Walter Gassmann, Paola Veronese, Steffen Heber

**Affiliations:** 1 Department of Computer Science, North Carolina State University, Raleigh, North Carolina, United States of America; 2 Department of Plant Pathology, North Carolina State University, Raleigh, North Carolina, United States of America; 3 Department of Plant Biology, North Carolina State University, Raleigh, North Carolina, United States of America; 4 Division of Plant Sciences, University of Missouri, Columbia, Missouri, United States of America; University of California, Los Angeles, United States of America

## Abstract

We report the results of a genome-wide analysis of transcription in *Arabidopsis thaliana* after treatment with *Pseudomonas syringae* pathovar *tomato*. Our time course RNA-Seq experiment uses over 500 million read pairs to provide a detailed characterization of the response to infection in both susceptible and resistant hosts. The set of observed differentially expressed genes is consistent with previous studies, confirming and extending existing findings about genes likely to play an important role in the defense response to *Pseudomonas syringae*. The high coverage of the *Arabidopsis* transcriptome resulted in the discovery of a surprisingly large number of alternative splicing (AS) events – more than 44% of multi-exon genes showed evidence for novel AS in at least one of the probed conditions. This demonstrates that the *Arabidopsis* transcriptome annotation is still highly incomplete, and that AS events are more abundant than expected. To further refine our predictions, we identified genes with statistically significant changes in the ratios of alternative isoforms between treatments. This set includes several genes previously known to be alternatively spliced or expressed during the defense response, and it may serve as a pool of candidate genes for regulated alternative splicing with possible biological relevance for the defense response against invasive pathogens.

## Introduction

The host-pathogen interaction between the model plant *Arabidopsis thaliana* and the bacterial foliar pathogen *Pseudomonas syringae* pathovar *tomato* is the result of a fascinating and ongoing co-evolutionary arms race [1], and the observed patterns of gene expression reflect the complex interplay between the immune system of the host and virulence factors of the pathogen. Exploration of this relationship at the level of mRNA transcription contributes to a detailed knowledge about the immune system of an important model organism, and can also serve as the basis for understanding similar interactions in economically important plant species.

The innate immune system of many plants contains two important layers of defense (see [2] for a detailed review). In the first layer, transmembrane pattern recognition receptors (PRRs) in plants respond to common classes of invasive biomolecules, including flagellin, bacterial cold shock proteins, and elongation factors, that are interpreted by the plant as indicators of the presence of potentially harmful microbes. Detection of these pathogen- or microbe-associated molecular patterns (PAMPs or MAMPs), occurs early during the infection and results in PAMP-triggered immunity (PTI). The second layer of the plant immune system occurs primarily inside the cell when plant disease resistance (R) proteins sense pathogen virulence effectors via mechanisms capable of distinguishing between self and nonself (or modified self). The resulting effector-triggered immunity (ETI) is, in general, faster and stronger than PTI, and often culminates in a hypersensitive response (HR) with associated local cell death in infected plant areas. Several examples of virulence effector – R protein interactions have been described (e.g. AvrRpm1 & RPM1, AvrRpt2 & RPS2) [3], [4].

Recent research suggests that alternative splicing (AS) can play a critical role in the defense response of plants [5]. For example, Dinesh-Kumar and Baker studied the tobacco *N* gene, a member of the Toll/interleukin-1 receptor (TIR) – nucleotide-binding (NB) – leucine-rich repeat (LRR) class of resistance genes. This gene encodes two alternatively spliced transcripts, with one variant lacking 13 out of 14 of the LRR repeat domains found in the longer transcript. Dinesh-Kumar and Baker showed that the truncated isoform is required for resistance to tobacco mosaic virus and that expression of this isoform increases 4–8 hours after infection [6]. Similarly, Zhang and Gassmann [7] found that alternative splicing of the *Arabidopsis* R gene *RPS4* is critically important for defense against *Pseudomonas*. However, since these studies have only targeted a small number of individual genes, it is unclear to what extent AS is involved in the immune response on a genomic scale. This paper attempts to overcome this gap. We report the results of a genome-wide analysis of transcription in *Arabidopsis thaliana* during a time course experiment involving treatment with *Pseudomonas syringae* pv *tomato*. The resulting data set contains over 1 billion paired-end RNA-Seq reads and provides evidence for a large number of previously unannotated AS transcripts in *Arabidopsis*, several of which occur in genes known to be involved in the defense response. In addition, differential expression of various known splice variants further supports an important role for AS in the immune response.

## Results

### Data set

We subjected healthy leaf tissue from 6 week old *Arabidopsis* seedlings of the Columbia (Col-0) accession to one of three treatments: 1) mock inoculation with 10 mM MgCl_2_ buffer, 2) inoculation with virulent *Pseudomonas syringae* pv *tomato* (*Pst*) DC3000 and, 3) inoculation with avirulent *Pst* DC3000 expressing the bacterial effector AvrRps4. Leaves subjected to infiltration with buffer only are expected to undergo significant changes in transcription in response to wounding [8], [9], and the mock treatment can therefore be used as a control to identify genes specifically regulated in response to pathogenic infection. Col-0 plants infected with the virulent *Pst* DC3000 strain are vulnerable to infection. In contrast, since this accession harbors the resistance gene *RPS4* which is capable of recognizing AvrRps4, Col-0 plants infected with avirulent *Pst* DC3000 are able to mount a defense response, conferring disease immunity.

Leaflets were harvested and pooled from at least 20 plants per treatment at 1, 6 and 12 hours post inoculation (hpi). The artificial inoculation was duplicated (biological replicates “A” and “B”); hence, the study has two experimental factors: treatment (MOCK, VIR, AVR) and time (1 hpi, 6 hpi, 12 hpi), for a total of 18 samples. Total RNA was extracted from each sample using the Qiagen Plant RNeasy Mini kit. The resulting RNA was then subjected to paired-end Illumina sequencing (2×75 nucleotide reads) at the David H. Murdock Research Institute (DHMRI, Kannapolis, NC) following the standard Illumina sample preparation and sequencing protocols. The experiment generated approximately 539 million read pairs; read counts and alignment statistics for each sample are provided in File S1. Sequences are available at the NCBI Sequence Read Archive (SRA, accession SRP010938).

### Alignment Results

Distributions for the FASTQ quality scores for each sample are available in the spreadsheets contained in File S2. After quality assessment with FastQC [10], it became clear that the sequence quality at the extreme ends of the reads was lower than the quality observed in the middle. Therefore, to enable high quality alignments, we used standard “end-trimming” software to preprocess the reads. In order to increase the efficiency and simplicity of subsequent downstream calculations, which rely on the pre-computation of a large k-mer table, we trimmed all reads to the same length, 66 nucleotides. Subsequently, we used bowtie [11] to generate unspliced alignments to the TAIR 10 transcripts. Approximately 80% (428.6 million) of the 538.7 million read pairs aligned to one or more TAIR 10 gene models. For the remaining 110.1 million reads pairs, we performed a spliced alignment to the TAIR 10 genome using TopHat [12]. Of these, approximately 2% (11.3 million read pairs) of the total either overlapped or were contained inside TAIR 10 genes, but did not have unspliced (bowtie) alignments to any of the annotated transcripts. In addition, a further 2% (11.2 million) of all read pairs aligned to intergenic regions, but did not map to any known genes.

To guarantee the highest possible data quality, we discarded read pairs that aligned to more than one gene, or contained one or more mismatches in their alignments. After this filtering step, we retained 317.9 million high quality transcriptome read pairs. See File S1 for details.

### Gene Expression Analysis

#### Expressed Genes

**Figure 1:**
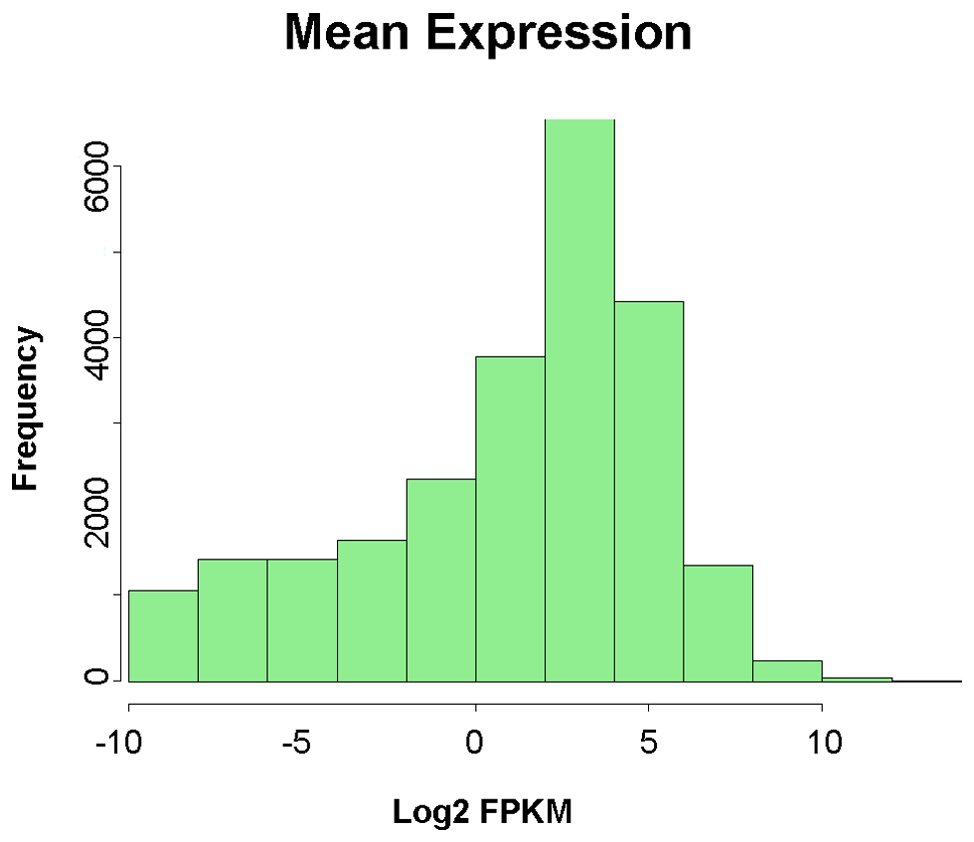
Distribution of gene expression. Shown is a histogram of the mean log_2_ IQ.OWLS FPKM expression levels for the 33,602 Arabidopsis genes in TAIR 10.

We computed the mean gene expression levels across all 18 samples. Figure 1 shows the distribution of the mean log_2_ FPKM values (fragments per kilobase of exon per million fragments mapped) [13] for the *Arabidopsis* genes in TAIR 10. FPKM values for multi-isoform genes were summed over the estimated IQ.OWLS transcript abundances (Methods S1). Among TAIR 10 genes, 72% (24,322 out of 33,602) had a mean FPKM above 0, indicating that a matching read pair was observed in at least one of the 18 samples. The median expression level was 0.8355 FPKM, and the maximum expression level was 7755 FPKM. File S3 contains the FPKM estimates for all samples.

#### Comparison to Microarray Data

**Figure 2:**
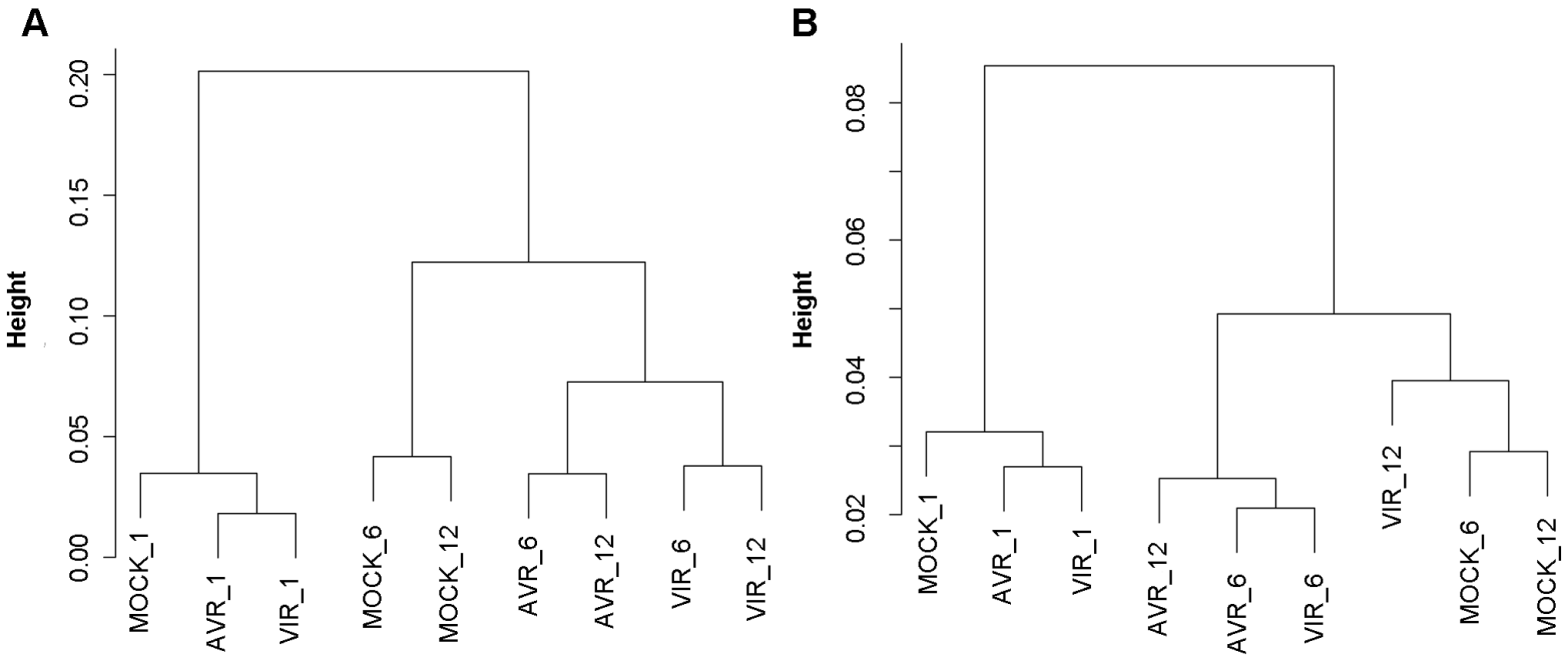
Hierarchical clustering of samples. A) By gene expression of defense response genes, B) by isoform expression of two isoform genes. Distances are 1 – Pearson correlations of log_2_ read counts.

We compared our RNA-Seq results to an Affymetrix ATH1 microarray experiment that examined the response of *Arabidopsis* to infection with various strains of *Pst* expressing different avirulent proteins including AvrRps4 [14]. This experiment includes differential gene expression data for avirulent and mock infected Col-0 plants at 6 hours post inoculation. We combined the RNA-seq reads of the two replicates, and measured the pairwise Pearson correlation of the resulting RNA-seq read counts for each of 4,515 genes that were differentially regulated in response to *Pst* infection in the microarray experiment. The resulting pairwise correlation matrix was converted to a distance measure (by subtracting from 1), and used to perform hierarchical clustering (Figure 2a). The samples are grouped first according to time after inoculation (early infection at 1 hpi versus late infection at 6 hpi and 12 hpi), and then by treatment, with the avirulent and virulent treatments generally more similar to each other than to the mock treatment. We obtained similar results when we measured the correlation of counts at the isoform level for the 4,318 TAIR10 genes with exactly 2 isoforms (Figure 2b). Subsequently, we examined genes that showed a significant fold change between avirulent and mock in the microarray experiment, and measured the correlation between these fold change values and the fold changes obtained from our own RNA-Seq experiment. At 6 hrs post inoculation, 3,075 genes showed significant differential expression in the microarray experiment, and for these genes, the correlation between the RNA-Seq and microarray fold changes was 0.81.

#### Differentially Expressed Genes

Files S4, S5, S6 contain ranked lists of differentially expressed genes (DEGs) in the pairwise comparisons between the AVR versus MOCK, VIR versus MOCK, and AVR versus VIR treatments at 1, 6 and 12 hours post inoculation. Differentially expressed genes were identified using both Cufflinks [13] and the EdgeR Bioconductor package [15]–[17]. The EdgeR package offers several variants of its testing procedure; we used both the EdgeR “classic” method, which is based on an exact test under the negative binomial model, as well as an alternate test based on a general linear models framework. In general, the EdgeR classic method produced the most conservative gene lists. On average, 85% of the genes in the EdgeR classic gene list also occurred in both the Cufflinks and EdgeR GLM gene lists. The other two methods produced longer gene lists, but with less agreement between the two methods; on average, only about 33% of the genes detected by at least one of these two methods were also detected by both methods. Due to the observed discrepancies between the three alternative methods, we adopted a “majority rules” strategy in which a gene is classified as differentially expressed if it is identified as such by at least two of the three methods. In the spreadsheets, genes that are classified as DEGs by all three methods are highlighted in green; genes identified by any two methods are highlighted in yellow.

**Figure 3:**
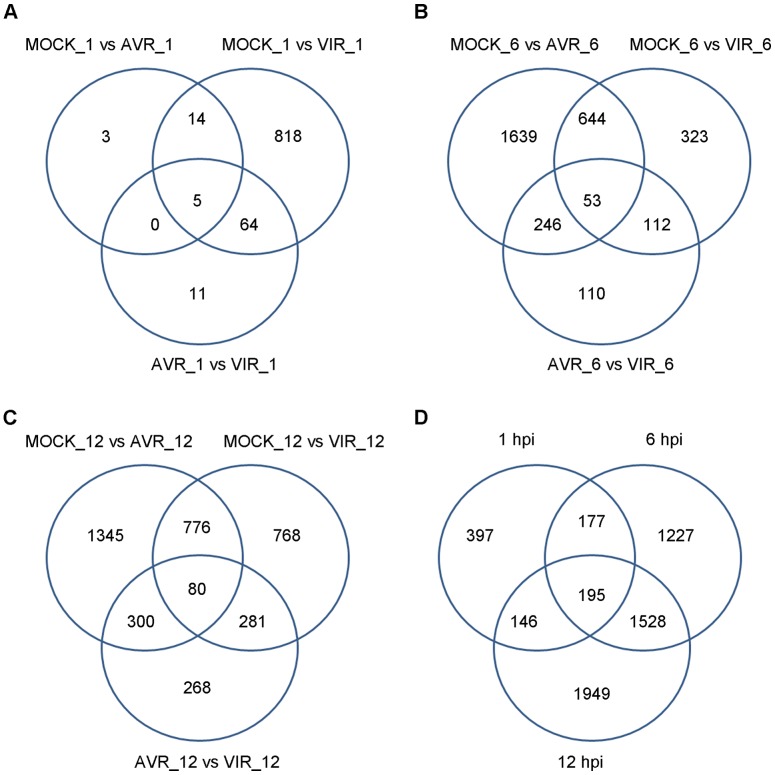
Differentially Expressed Genes (DEG). A) By treatment comparison at 1 hpi, B) by treatment comparison at 6 hpi, C) by treatment comparison at 12 hpi, and D) all treatment comparisons, by time point.

The number of differentially expressed genes detected increased steadily during the course of the infection. For example, for the mock versus virulent comparisons, 901, 1132 and 1905 genes were identified by at least two methods at 1, 6, and 12 hpi, respectively (Table 1, Figure 3). This behavior reflects the expected dynamics for the induction of the *Arabidopsis* defense response (Quirino & Bent, 2003). However, the relatively small number of differentially expressed genes at 1 hpi is also likely due, in part, to a lower power to detect differential expression given the smaller total number of RNA-Seq reads sequenced at this time point. In addition, as indicated in Figure 3A and File S4, there were considerably more DEGs detected for the MOCK vs. VIR treatment at 1 hpi relative to the other two comparisons (MOCK vs. AVR and AVR vs. VIR). Our data indicate that many of the early gene expression levels observed in AVR are intermediate to those in the MOCK and VIR treatments. For example, in 796 out of 901 (88%) of the 1hpi DEG genes found in the VIR vs. MOCK comparison, the observed FPKM for the AVR treatment was in between the FPKMs from VIR and MOCK.

**Table 1:** Summary of DEG, DEI and DIR.

	1 hpi			6 hpi			12 hpi		
**A) DEG**	1 method significant	2 methods significant	all significant	1 method significant	2 methods significant	all significant	1 method significant	2 methods significant	all significant
***avirulent*** ***vs mock***	198	22	1	3861	2582	1718	8392	2501	984
***virulent*** ***vs mock***	2481	901	283	2636	1132	720	6056	1905	699
***avirulent*** ***vs virulent***	399	80	13	1382	521	133	1943	929	380
**B) DEI**	1 method significant	2 methods significant	all significant	1 method significant	2 methods significant	all significant	1 method significant	2 methods significant	all significant
***avirulent*** ***vs mock***	54	3	0	1167	342	206	2921	279	112
***virulent*** ***vs mock***	962	113	19	901	185	110	2183	283	106
***avirulent*** ***vs virulent***	274	18	1	207	33	6	723	125	35
**C) DEI +** **DIR**	1 method significant	2 methods significant	all significant	1 method significant	2 methods significant	all significant	1 method significant	2 methods significant	all significant
***avirulent*** ***vs mock***	0	0	0	38	20	12	75	27	16
***virulent*** ***vs mock***	24	9	3	48	24	18	104	58	23
***avirulent*** ***vs virulent***	10	3	0	10	3	0	21	8	2

The resulting DEG lists include several genes that are well-characterized markers of early and late defense responses against *Pst*, as well as key regulatory components of the *Arabidopsis* innate immune system. For example, At2g19190 (*FRK1*), a PAMP-responsive gene which encodes a flagellin receptor-like kinase that participates in the innate immune response to infection, has previously been shown to be up-regulated within 30 minutes of infection [18], [19]. At 1 hpi this gene exhibited a nearly 8-fold increase in the virulent versus mock and avirulent versus mock treatments in our experiment. Likewise, the gene At4g23550 (*WRKY29*) was also up-regulated at 1hpi, in accordance with previous studies of the innate immune response [19]. At 6 and/or 12 hours post inoculation, important markers for infection and defense, including *PR1* (At2g14610), *PAD4* (At3g52430) and *EDS1* (At3g48090) [20] are up-regulated in mock versus treated samples, and the gene lists for treated samples at 6 and 12 hpi include numerous TIR-NB-LRR resistance genes, transcription factors and stress-response genes.

We performed a GO-term enrichment analysis of the set of all genes differentially regulated, according to the majority vote, at 1, 6 and 12 hpi, resulting in 157, 380, and 388 significant terms, respectively (0.10 FDR-corrected p-value). Detailed lists are provided in Files S4, S5, and S6 and visual summaries created using the AgriGO tool [21] are available in File S10. The spreadsheets for each time point include lists of GO terms significant for the individual pairwise comparisons as well as GO terms enriched for the combined set of all genes differentially expressed in at least one of the comparisons. Overall, the gene lists were highly enriched for relevant terms including ‘defense response’, ‘innate immune response’, ‘response to bacterium’, ‘programmed cell death’, ‘signal transducer activity, ‘transcription factor activity’, and ‘transmembrane receptor activity’.

#### Differentially Expressed Isoforms

The TAIR10 gene set contains 5,885 genes with multiple annotated isoforms. For each gene in this set of 5,885 multi-isoform genes, we also tested for differential expression at the level of individual isoforms, once again employing several alternative approaches to generate lists of differentially expressed transcripts (see Methods). The first two approaches consider only reads that align to the unique regions in each transcript. These read counts are then tested for differential expression using the same EdgeR ‘classic’ and GLM frameworks we previously used to compare read counts at the whole gene level. In addition, we also used the Cufflinks software to test for differential isoform expression. For each comparison, the EdgeR classic method produced the smallest transcript list and the Cufflinks method produced the largest transcript list. For example, for the mock versus virulent comparison at 1 hpi, the three methods identified 23, 230 and 841 transcripts. Among the 23 transcripts identified by EdgeR classic, 19 (91%) also occurred in both of the other two lists. Approximately 47% of the 230 genes identified by EdgeR GLM also occurred in the Cufflinks list.

**Figure 4:**
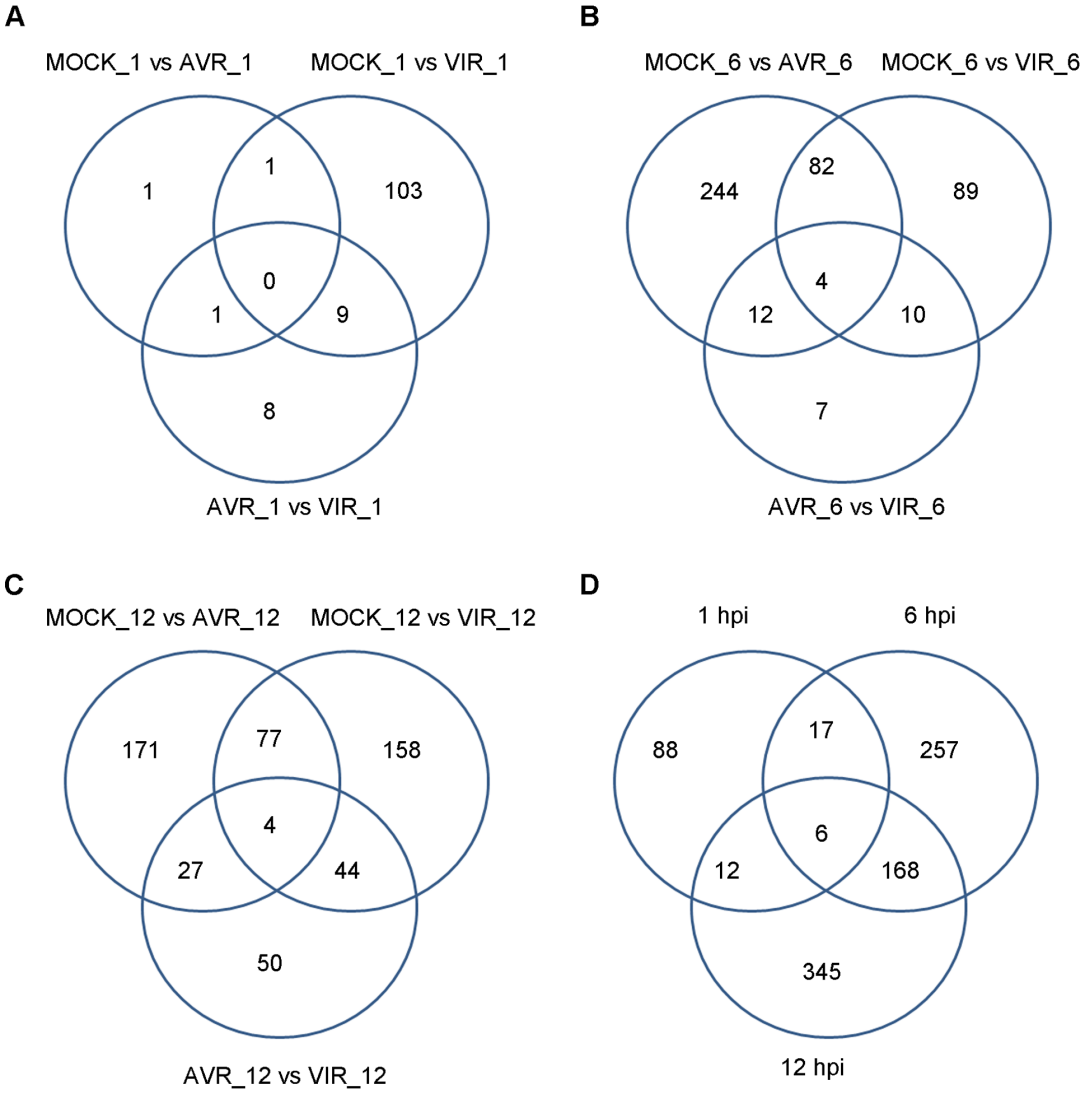
Differentially Expressed Isoforms (DEI). A) By treatment comparison at 1 hpi, B) by treatment comparison at 6 hpi, C) by treatment comparison at 12 hpi, and D) all treatment comparisons, by time point.

The resulting lists of differentially expressed isoforms (DEI) are provided in Files S7, S8, and S9. Genes identified by all three methods are highlighted in green, and genes identified by any two methods are highlighted in yellow. As in the case of differential expression computed at the gene level, the number of differentially expressed isoforms detected increased during course of infection. For example, for the mock versus virulent comparisons, 113, 185 and 283 genes were identified by majority vote at 1 hpi, 6 hpi, and 12 hpi, respectively (Table 1, Figure 4).

We also tested for GO term enrichment in the differentially expressed isoforms and identified several relevant terms including many of the same terms identified at the whole gene level. The GO terms identified were very similar to the terms identified for DEG genes. However, there were fewer total significant GO terms for the set of differentially expressed isoforms – 15, 15, and 36 terms at 1 hpi, 6 hpi and 12 hpi, respectively. The terms identified clearly suggest the presence of a strong defense response and include a variety of defense-related terms such as “response to stress”, “defense response”, “immune response” and “response to biotic stimulus”. Also, as is the case for DEG, the significant terms suggest that many of the DEI genes are localized in the chloroplast, an organelle known to play an important role in the mediation of plant innate immunity [22], [23]. The full lists of identified GO terms are provided along with the lists of differentially expressed genes in the Supporting Information Files. In addition, visual summaries are available in File S10.

### Alternatively spliced transcripts

Differential isoform expression can arise both as a result of gene-level regulatory signals (e.g. transcription factors, chromatin folding, etc.) and also in response to regulatory signals that affect individual transcripts, or groups of transcripts, including alternative promoters, and splicing factors. In order to identify genes with isoforms whose expression might be regulated by transcript-specific regulatory signals, we sought to quantify the ratios of the individual transcript isoforms for each gene, and to then identify significant changes in these expression ratios in response to our experimental factors.

For each of the 5,885 multi-isoform genes, we first estimated the percentage of each transcript isoform as a percentage of the total expression for the gene. These percentages were computed using two different methods for isoform quantification: IQ.OWLS (Methods) and Cufflinks. We performed qRT-PCR validations for 20 of the 2-isoform genes, using multiple RNA samples, for a total of 96 qPCR reactions. File S12 contains the results. Overall, we observed a Pearson correlation of 0.74 between the transcript isoform percentages computed using IQ.OWLS and the percentages obtained from qPCR. The correlation between the Cufflinks percentages and qPCR was 0.51; however, the relatively poor performance of Cufflinks was driven by a single outlier transcript. When this transcript is removed, the correlation between Cufflinks and qPCR increases to 0.69, and the correlation between Cufflinks and IQ.OWLS increases from 0.78 to 0.96.

**Figure 5:**
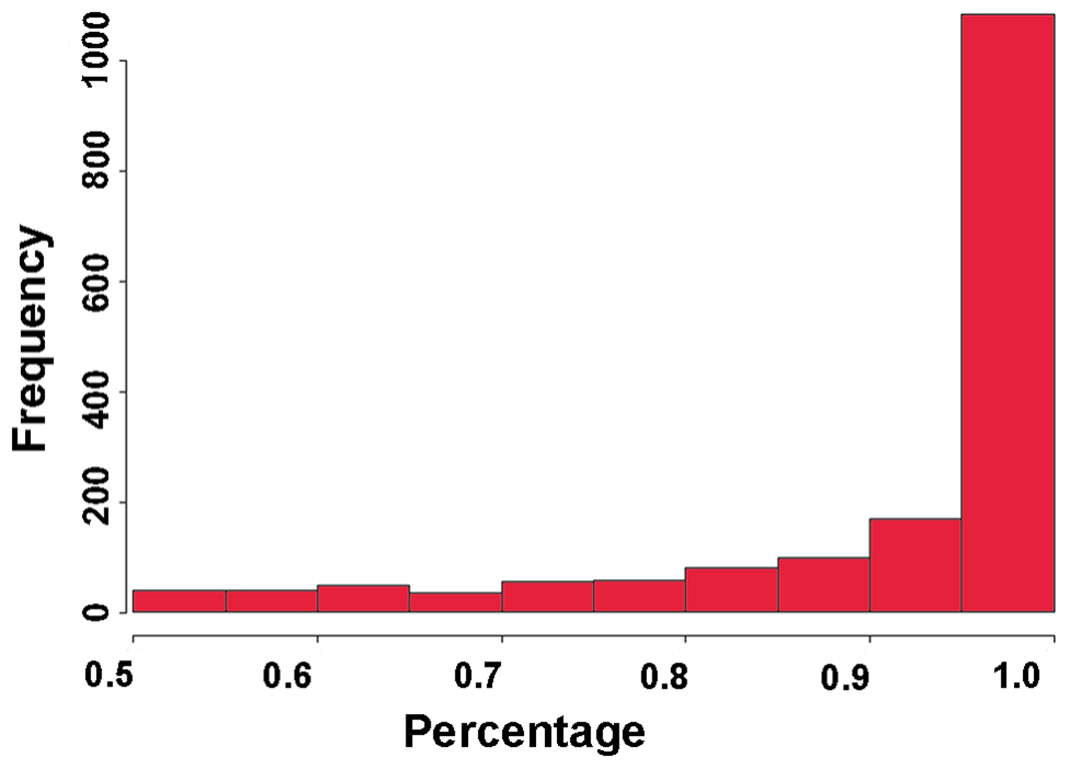
Major isoform percentages. Distribution of the IQ.OWLS estimates for the major (most highly expressed) isoform in 2-isoform genes expressed with at least 500 reads (1,695 out of 4,318 genes) in mock treated leaves at 6 hpi.

Figure 5 shows the distribution of the resulting IQ.OWLS estimates for the most highly expressed isoform in a subset of the 2-isoform genes in mock treated leaves at 6 hpi. Since the accuracy of isoform expression estimates is expected to increase with read coverage, Figure 5 includes only those genes expressed with at least 500 read pairs (1,695 out of 4,318 genes). As shown in the figure, most of these 2-isoform genes have a clearly dominant isoform, an observation that is in agreement with previously reported results [24]. Nevertheless, many of these genes also showed a clearly measurable expression signal for the minor isoform as well. For approximately 33% of the genes, the minor isoform made up 5% or more of the mixture; for 24% of genes, the minor isoform made up 10% or more of the mixture; and, for 11% of genes, the minor isoform made up 25% or more of the mixture.

**Figure 6:**
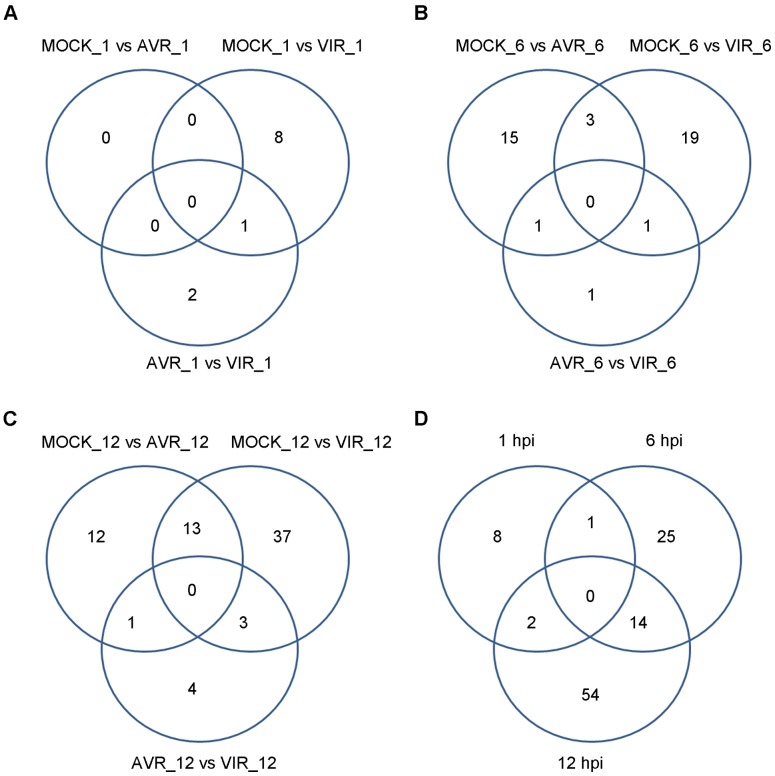
Differentially alternatively spliced (DEI + DIR) isoforms. A) By treatment comparison at 1 hpi, B) by treatment comparison at 6 hpi, C) by treatment comparison at 12 hpi, and D) all treatment comparisons, by time point.

We next computed the change in transcript isoform mixture percentage for each isoform across treatments and identified genes with differential isoform ratios (DIR) in cases where the 95% confidence interval for this difference did not contain zero (Methods S1). Differentially expressed isoforms (DEI transcripts) which also exhibit DIR are highlighted in red in Files S7, S8, and S9. (See also: Table 1, Figure 6).

**Figure 7:**
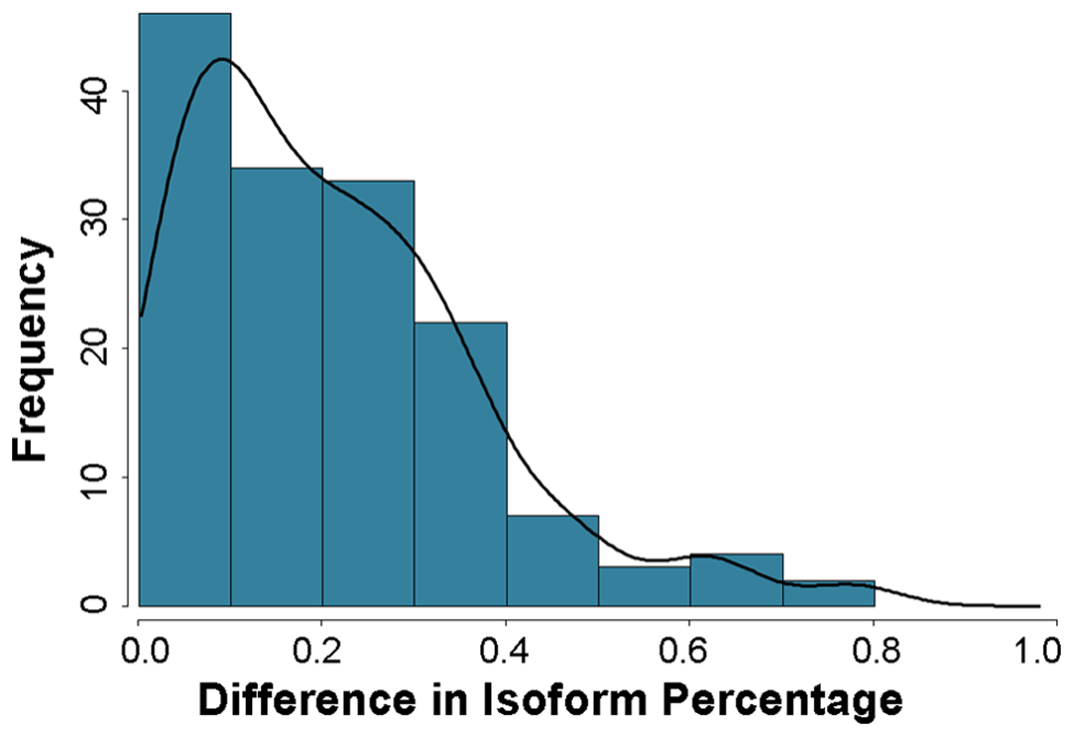
Transcript isoform percentage changes among the set of DEI + DIR transcripts. Most of the differentially expressed transcript did not show large changes in the relative frequency of the dominant isoform, with few transcripts exhibiting “switch-like” expression changes.

Figure 7 shows the distribution for these isoform mixture percentage changes among the set of all DEI + DIR transcripts. The median difference was approximately 18%. The figure reveals that there apparently are not a large number of transcripts displaying “switch-like” behavior between treatments. For example, only about 6% out of the 151 DEI+DIR transcripts were detected as expressing a change in mixture percentage of 50% or more. However, it is important to recognize that the observed measurements are from tissue-level mRNA extractions pooled across leaves harvested from several individual plants. It is therefore impossible to observe whether or not switch-like regulation occurs at the cell-level.

The full list of DEI+DIR transcripts is available in File S11. Of special interest were a subset of these genes, 29 in all, which exhibited at least a 10% change in mixture percentage in isoforms where the observed splicing events occur in the vicinity of or alter an annotated pFAM protein domain [25]. These genes, listed in Table 2, contain several genes previously shown to play important roles in the defense response. File S111 contains additional annotation information for these genes, along with PubMed references to relevant publications linked to these genes in the TAIR 10 database. Interestingly, only 11 of the 29 genes (38%) were also identified as DEG in our experiment; the remaining 18 genes may not have been detected as differentially expressed in a standard gene-level analysis.

**Table 2:** Significant DEI + DIR events found in the vicinity of a pFAM domain.

Time	Transcript AGI		Gene Name	Short_Description	DEG?
**1 hpi**	At2g38170.3	*CAX1*		cation exchanger 1	0
	At4g39270.2			Leucine-rich repeat protein kinase family protein	0
	At4g39270.1			Leucine-rich repeat protein kinase family protein	1
	At5g46110.2	*APE2, TPT*		Glucose-6-phosphate/phosphate translocator-related	0
	At1g51620.1			Protein kinase superfamily protein	1
**6 hpi**	At4g35770.2	*SEN1*		Rhodanese/Cell cycle control phosphatase superfamily protein	0
	At5g41610.1	*CHX18*		cation/H+ exchanger 18	1
	At5g05580.1	*FAD8*		fatty acid desaturase 8	1
	At4g04830.1	*MSRB5*		methionine sulfoxide reductase B5	1
	At5g28770.3			bZIP transcription factor family protein	1
	At4g29210.2	*GGT4*		gamma-glutamyl transpeptidase 4	0
	At1g67300.2			Major facilitator superfamily protein	0
	At4g37980.2	*CAD7, ELI3, ELI4*		elicitor-activated gene 3–1	0
	At2g14560.2	*LURP1*		Protein of unknown function (DUF567)	1
**12 hpi**	At2g46370.4	*JAR1*		Auxin-responsive GH3 family protein	1
	At1g67300.2			Major facilitator superfamily protein	0
	At5g43910.2			pfkB-like carbohydrate kinase family protein	1
	At5g41610.1	*CHX18*		cation/H+ exchanger 18	1
	At4g29210.2	*GGT4*		gamma-glutamyl transpeptidase 4	0
	At4g19040.2	*EDR2*		ENHANCED DISEASE RESISTANCE 2	0
	At2g20740.3			Tetraspanin family protein	0
	At5g07440.2	*GDH2*		glutamate dehydrogenase 2	1
	At4g37980.2	*CAD7, ELI3, ELI4*		elicitor-activated gene 3-1	0
	At4g32440.1			Plant Tudor-like RNA-binding protein	0
	At5g14200.1	*IMD1*		isopropylmalate dehydrogenase 1	1
	At4g37980.2	*CAD7, ELI3, ELI4*		elicitor-activated gene 3-1	0
	At5g17760.2			P-loop containing nucleoside triphosphate hydrolases superfamily protein	1
	At5g41610.1	*CHX18*		cation/H+ exchanger 18	1
	At4g07410.2	*PCN, POPCORN*		Transducin family protein/WD-40 repeat family protein	0
	At5g46110.2	*APE2, TPT*		Glucose-6-phosphate/phosphate translocator-related	1
	At4g29210.2	*GGT4*		gamma-glutamyl transpeptidase 4	0
	At1g67300.2			Major facilitator superfamily protein	0
	At2g28550.3			related to AP2.7	0
	At5g14200.1	*IMD1*		isopropylmalate dehydrogenase 1	0
	At5g07440.2	*GDH2*		glutamate dehydrogenase 2	1
	At2g16710.1			Iron-sulphur cluster biosynthesis family protein	0
	At4g23330.1				0
	At3g54840.2	*ARA, RABF1*		Ras-related small GTP-binding family protein	0
	At5g60590.2			DHBP synthase RibB-like alpha/beta domain	0
	At1g17130.2	*DUF572*		Family of unknown function (DUF572)	0
	At4g39100.2	*SHL1*		PHD finger family protein/bromo-adjacent homology (BAH) domain-containing protein	0

Our results also reveal that dependent on the structure of the investigated locus, several hundred to several thousands of read pairs are required to get tight confidence intervals for the mixture percentages. The accuracy of quantification is also limited by the completeness of the transcript catalog used for quantification. This is a serious problem since our data show evidence for many new transcripts and alternative splicing events.

### Novel Splicing Events

Over 90% of the expressed genes (23,385 out of 25,619) had at least one inconsistent read pair which aligned to the gene region, but not to any known transcript in at least one of the 18 samples sequenced; almost 65% of the expressed genes had 5 or more inconsistent read pairs in at least one sample. Furthermore, using conservative detection criteria, approximately 40% (10,224/25,619) of expressed genes showed evidence for novel alternative splicing events (Methods S1). In addition, 51% of expressed genes (13,073 out of 25,619) had 2 or more reads extending the annotated 3′ UTR, while 56% of expressed genes (14,297 out of 25,619) had 2 or reads extending the annotated 5′ UTR (File S13.).

We detected 84% (107,144 out of 128,271) of the known TAIR10 splice sites with at least one read pair, and 82% (104,567 out of 128,271) were detected with 2 or more independent (different start site) read pairs. In addition, we found 57,360 novel splice junctions with at least one read, 45% (25,864) of which were represented by 2 or more independent reads. See File S14 for details.

**Figure 8:**
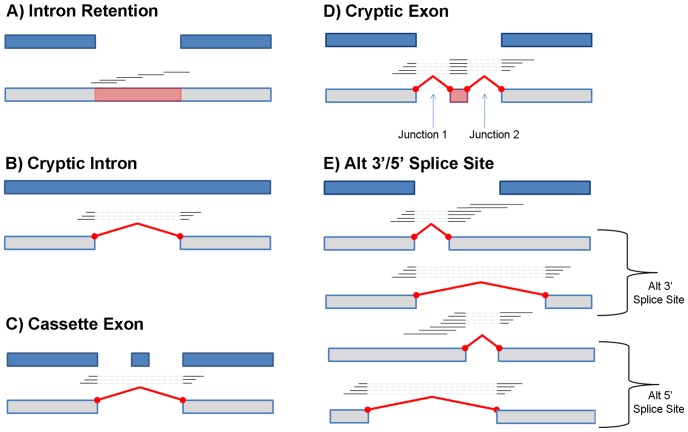
Five different types of novel AS events. The events shown are intron retention, cryptic intron, cassette exon, cryptic exon, and alternative 3′/5′ splice site.

We mined our data set for 5 different types of AS events: intron retention, cryptic intron, cassette exon, cryptic exon, and alternative 3′/5′ splice site (Figure 8). All of the novel alternative splicing events that we report use splice junctions that are supported by a minimum of two read pairs with different read coordinates. Furthermore, each novel event type has specific architectural constraints that must be satisfied. The details of the procedures used to classify each of these events are described in Methods S1.

We counted novel AS events, and AS events that are already represented in the TAIR 10 transcript catalog, arbitrarily choosing the first listed transcript (usually the one with the “.1” extension) as the primary isoform. Surprisingly, we found that more than 44% of the multi-exon genes showed evidence for novel AS events (see Table 3 for a summary).

**Table 3:** Summary of detected AS events.

Event Type	Known Events Detected/Known Events	Novel Events Detected	Genes with Novel Event
Splice Junction	104,567/128,271 (82%)	25,864	10,400
Intron Retention	738/1,312 (56%)	14,934	7,755
Cryptic Intron	730/1,222 (60%)	2,508	1,408
Alt 3′/5′ Splice Site	1,657/2,740 (60%)	8,886	5,344
Cassette Exon	127/206 (62%)	491	477
Cryptic Exon	107/374 (29%)	76	73

The complete set of novel candidate events is available for interactive exploration at the following url: http://152.14.14.56/cgi-bin/gbrowse/EAGER-Novel-AS/#search. In following sections we discuss each AS event type separately.

#### Novel Intron Retention

**Figure 9:**
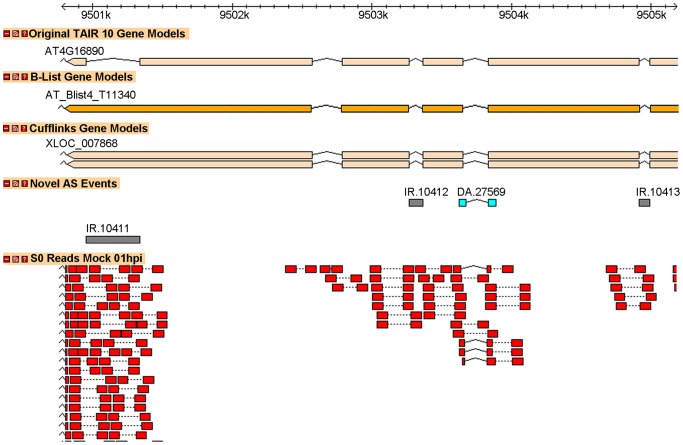
Novel intron retention event in gene At4g16890. This gene “encodes a Toll Interleukin1 receptor-nucleotide binding-Leucine rich repeat-type resistance gene (TIR-NB-LRR-type) involved in the salicylic acid-dependent defense response pathway. Mutant plants constitutively express pathogenesis-related (PR) genes and are pathogen resistant. Resistance signaling in snc1 requires EDS1, MOS3 and PAD4”. In this case, the event is described in [34] and corresponds to a TAIR 10 “B-List” gene.

We identified 14,934 novel intron retention events (Figure 8a) in 7,755 distinct genes; this was the most common of the observed splicing event types. Several of the events occurred in all 18 samples, including the example shown in Figure 9. For 911 genes, novel intron retentions were observed in both replicates for one or more of the avirulent and/or virulent treatments, but not in any of the mock treated samples. Several genes which are known to play important roles in the *Arabidopsis* defense response to Pseudomonas are affected, including: At1g80840 (*WRKY DNA-binding protein 40*), At2g19190 (*FLG22-induced receptor-like kinase 1*), At2g04450 (*nudix hydrolase homolog 6*), and At3g48090 (*EDS1*). Details of the procedure used to identify intron retentions are described in Methods S1. The full list of intron retention events is available in File S15.

The GO terms associated with novel intron retention events indicate that many of these genes may be important for the defense response. For example, the gene list was highly enriched for relevant GO terms including “response to other organism”, “response to bacterium”, “defense response”, “immune response”, and “plant-type hypersensitive response”. We identified 358 significant GO terms in total; the full list appears in File S15 and a visualization of the significant terms is provided in File S10.

Additional bench work will be required to determine which of these intron retention events are biologically important. We expect that some of the detected events may originate from unspliced transcripts, or splicing errors that are not actually functionally relevant. However, previous studies have indicated that intron retention is the most common type of alternative splicing in *Arabidopsis* [26], [27], and that in many cases these events play critical functional roles, including regulation by nonsense-mediated decay.

#### Cryptic Introns

**Figure 10:**
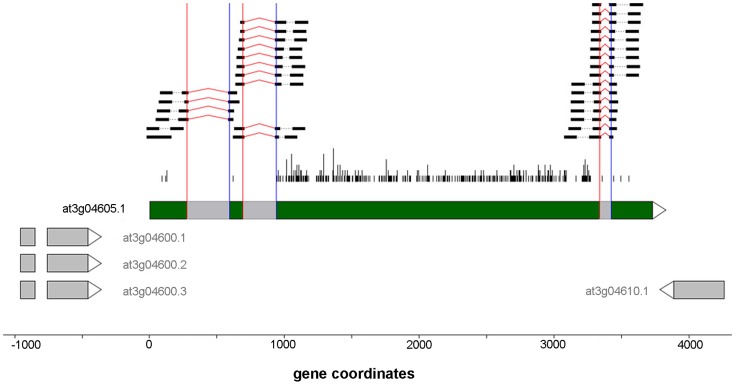
Novel cryptic intron event in gene At3g04605, which has the TAIR 10 annotation “transposable element gene”. In the figure the red and blue vertical lines indicate the 5′ and 3′ splice boundaries of the putative introns (shown in gray). S_0_ reads are shown at the top; start positions of non-S0 reads shown by vertical black bars.

Our analysis identified 2,508 novel cryptic intron events (Figure 8b) occurring in 1,408 distinct genes. When these events were ranked according to the total number of supporting reads, the majority of the top scoring candidates (e.g., 9 out of the top 10 events, by junction read count) occurred in single-exon transposable element genes and/or pseudo genes. Figure 10 displays one of the genes (transposable element gene At3g04605) where well supported cryptic introns were observed in all 18 samples. While it is difficult to speculate on a possible functional role for these events, recent research has revealed that in many cases, pseudogenes and transposons appear to be under purifying selection and can play apparent regulatory roles through the RNA interference pathway [28]. Alternatively, some of the detected events may simply reflect wide-spread dysregulation of transcription and splicing due to the pathological diseased state of the observed tissues.

We once again focused specifically on instances where the candidate novel cryptic intron events occurred only in the avirulent and/or virulent treatments but not the mock treatment. We identified 114 such events occurring in 94 distinct genes. In contrast to the cryptic intron events that were identified in all or most of the samples, these treatment-specific events were not dominated by transposons and pseudogenes. Instead, as was the case for novel intron retention events, the corresponding genes were enriched for relevant GO terms: “response to other organism”, “multi-organism process”, and “response to biotic stimulus”. (See Files S16 and S10 for details.)

#### Alternative 3′ or 5′ Splice Site

**Figure 11:**
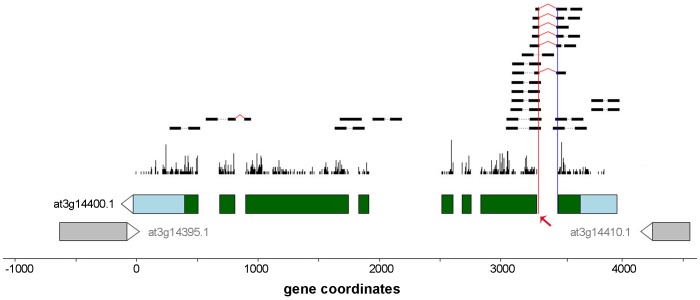
Candidate alternative 3′ splice site (indicated by red arrow) in gene At3g14400 “ubiquitin-specific protease 25” . This alternative splicing event, which introduces a frame-shift mutation, is observed in both replicates of avr and vir treatment at 6 and 12 hpi, but not in mock treatments.

We identified 8,886 novel alternative 3′/5′ splicing events (Figure 8e) among 5,344 distinct genes. 5,926 of these events were alternative 3′ splice site and the remaining 2,960 events involved alternative 5′ splice sites. Genes having events unique to the avirulent and/or virulent treatments were enriched for functionally relevant categories (e.g. “response to other organism”, “defense response”, etc.) suggesting a possible functional role for some of these events, see Files S17 and S10 for details. Figure 11 shows an example of one of the events with a large number of supporting reads. The figure displays an alternative 3′ splice site in gene At3g14400 “ubiquitin-specific protease 25”. This alternative splicing event, which introduces a frame-shift mutation, is observed in both replicates of the avirulent and virulent treatments at both 6 and 12 hpi, but not in the corresponding mock treatments.

#### Cassette Exons

**Figure 12:**
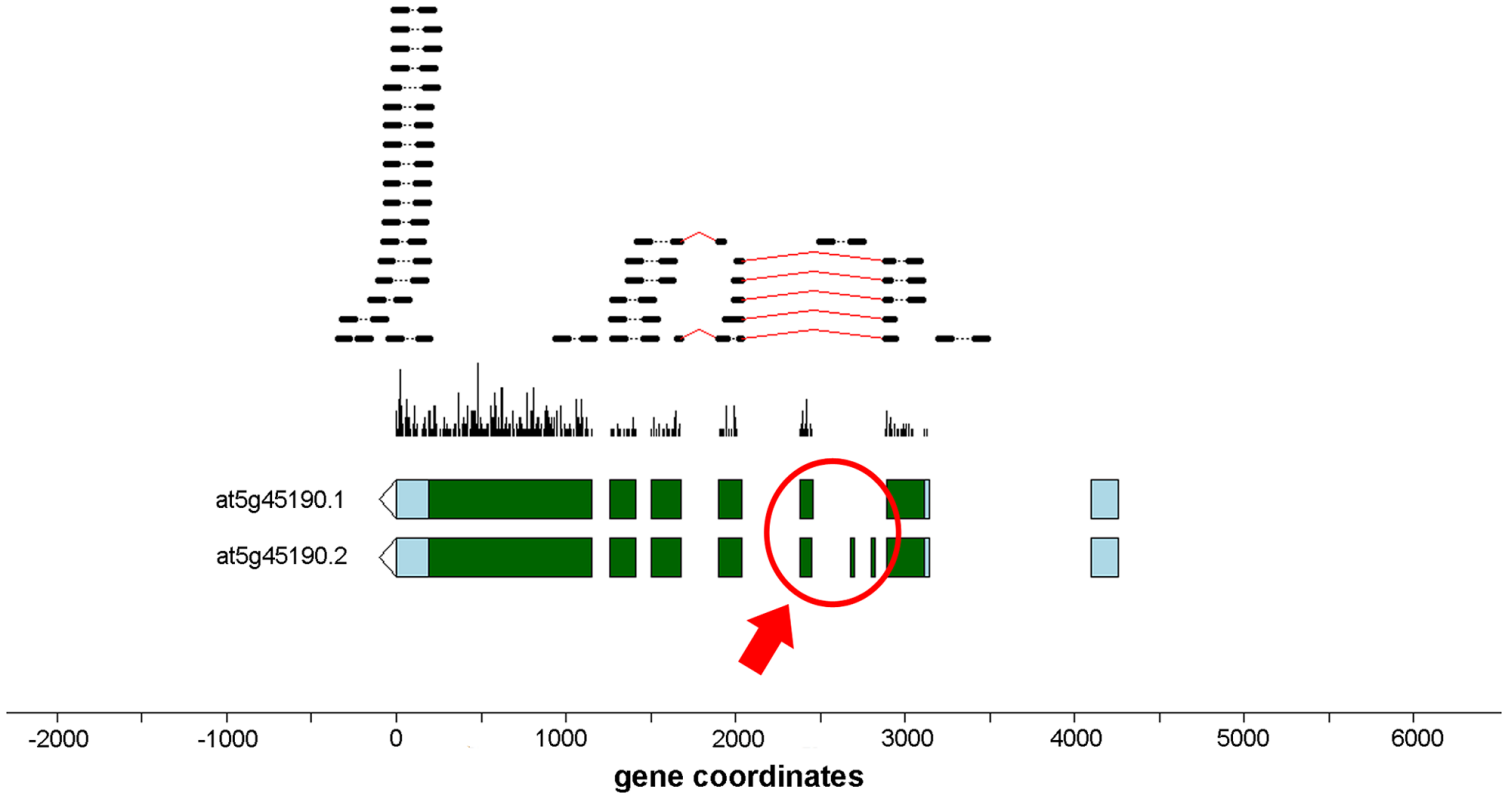
Novel cassette exon event in gene At5g45190 “Cyclin T partner CYCT1;5”. Putative cassette exon(s) are indicated by the red arrow. This gene, which has been previously shown to play an important role in infection with Cauliflower mosaic virus [29], has a TAIR 10 B-List transcript which confirms the indicated exon skipping event.

In comparison to the other event types, novel cassette exon events (Figure 8c) and cryptic exon events (Figure 8d) were relatively rare. However, we still detected evidence for 491 novel cassette exons among 477 distinct genes. Furthermore, 28 genes had events that were detected only in the avirulent and/or virulent treatments but not in the mock samples. Figure 12 displays an example of a candidate event which was observed only in the AVR and VIR samples in the gene At5g45190, “cyclin T partner CYCT1;5”. CYCT1;5 knockout mutants have been previously shown to be highly resistant to the Cauliflower mosaic virus [29]. The figure indicates that there are several reads with splice junctions that are not consistent with any of the annotated transcripts, but which appear to join the second and fourth exons of transcript At5g45190.1, or equivalently the second and sixth exons of transcript At5g45190.2. The full listing of cassette exon events is available in File S18.

#### Cryptic Exons

**Figure 13:**
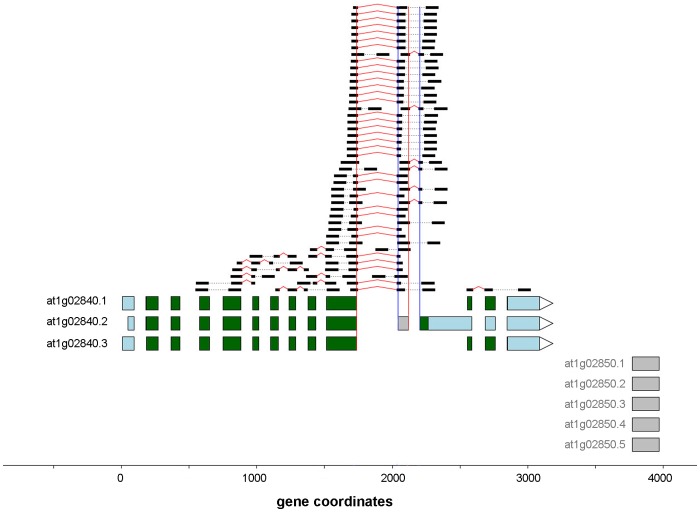
Novel cryptic exon event in gene At1g02840, splicing factor SR1. In the figure the red and blue vertical lines indicate the 5′ and 3′ splice sites and the grey box indicates the candidate cryptic exon.

We detected a total of 77 cryptic exon events (Figure 8d) in 74 distinct genes. The relative rarity of these events in part reflects the greater number and specificity of RNA-Seq reads required to detect a cryptic exon event. We require both splice junctions to be covered with at least 2 independent reads, plus an average coverage of 2 reads along the entire candidate exon. In contrast, other event types, such as cryptic introns and cassette exons are detectable from a single splice junction (see Methods S1). Three genes showed events that were detected only in the avirulent and/or virulent treatments but not control. Figure 13 shows a novel cryptic exon identified in the splicing factor SR1. The full listing is available in File S19.

### Novel Genes and Transcripts

We used Cufflinks to assemble novel transcripts from the RNA-Seq data. This resulted in 22,212 novel transcripts, including 165 unknown, intergenic transcripts, promising candidates for novel genes. Novel transcripts were more commonly observed in genes that already have more than one annotated isoform in TAIR 10. Approximately 25% of the single isoform genes in TAIR 10 produced evidence for novel transcripts, compared to 60% of the TAIR 10 genes with multiple transcripts. The Cufflinks gene models are available in File S20 and at: http://152.14.14.56/cgi-bin/gbrowse/EAGER-Novel-AS/#search.

**Figure 14:**
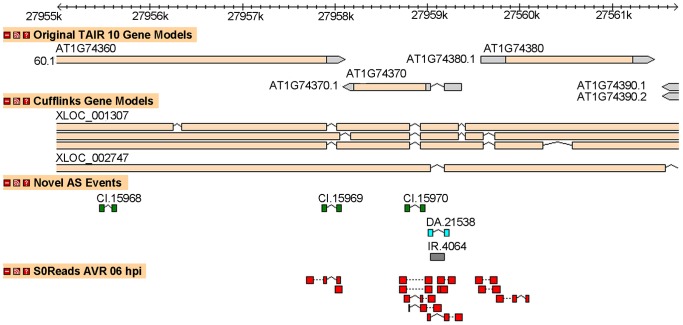
Novel events versus Cufflinks gene models.

The splicing events detected by Cufflinks and the events detected using the procedures described above are not always in agreement. For example, Figure 14 shows that the two methods agree for 2 out of the 3 pictured novel cryptic intron events (CI.15969 and CI.15970), and for a novel intron retention event (IR.4064). However, using the procedure previously described, we also identified an additional cryptic intron event (CI. 15968) as well as a novel alternative 3′ splice site (DA.21538). Conversely, the proposed Cufflinks gene models also imply several unique events.

## Discussion and Conclusions

We have provided a detailed characterization of the *Arabidopsis thaliana* transcriptional response to *Pseudomonas syringae* infection in both susceptible and resistant hosts. Previous microarray-based studies of this host-pathogen interaction have been limited to a subset of well annotated *Arabidopsis* genes, without taking splice variants into account. For example, compared with a recent study [14] based on results from the Affymetrix ATH1 GeneChip, we report here the expression levels for all 33,602 genes annotated in TAIR 10, including 11,371 genes not included in the microarray platform. Furthermore, in contrast to studies performed with microarrays, RNA-Seq based research is not limited to the set of known transcripts for which probesets have been designed. This is an important distinction, since our results indicate that the set of genes and transcripts cataloged in the TAIR 10 database represents only a small subset of all *Arabidopsis* transcripts. We provide evidence for 165 unannotated intergenic transcripts, potentially pseudogenes, or novel genes. In addition, approximately 25% of all annotated TAIR 10 single-isoform transcripts and 60% of TAIR 10 multi-isoform transcripts produced evidence for novel transcripts. Consistent with previous research [24], we observed that the majority of two isoform genes expressed one clearly dominating major isoform (Figure 5). In about 75% of the two isoform genes, the major isoform made up more than 90% of the mixture. On the other hand, almost all of the two isoform genes exhibit some expression of both isoforms, and for approximately 11% of the two-isoform genes, the minor isoform was expressed as 25% or more of the mixture.

We also detected a complex layer of so far unknown splice variants. In concordance with previous studies, the most common novel splicing event types were retained introns (14,934 distinct events) and alternative donor and acceptor splice sites (8,886 distinct events), but we also detected evidence for 2,508 cryptic introns, 491 candidate cassette exons and 76 cryptic exons. These data are summarized in Table 3, and the evidence for these events along with the 22,212 novel transcripts proposed by Cufflinks are available online. To validate our predictions we cross-checked these novel splicing events with TAIR's B-list [30], a list of 1,737 putative but highly supported alternative transcripts (1,640 provided a cross-reference to an existing TAIR10 gene) that currently are not included in the TAIR 10 transcriptome, and have not been used in our analysis. In total, 298 novel splicing events could be validated by B-list transcripts. This corresponds to almost 20% of the B-list transcripts. In general, a novel alternative splicing event was more likely to occur in the B-list if it was detected in multiple RNA-Seq samples, and showed a higher read coverage on average. For example, in the case of the 26 confirmed cryptic intron events, the median number of samples supporting the event was 6 (out of 18). For unconfirmed events, the median number of supporting samples was 2. Similarly, for the confirmed intron retention events, the median number of supporting samples was 14 compared to 2 for the unconfirmed events. Confirmed events were more highly expressed on average; for confirmed intron retention events, the median coverage was 13.5 reads (per sample) compared to 3.6 reads for the set of all intron retention events. (Data not shown.).

While some of the predicted intron retention events might represent partially unspliced transcripts, our results are consistent with previous findings indicating that intron retention is the most common form of alternative splicing in *Arabidopsis* [26]. Furthermore, it has been postulated that intron retention may play an important role in the regulation of the *Arabidopsis* defense response via nonsense-mediated decay (NMD), although the exact mechanisms are still unclear. For example, in [31] the authors hypothesize that NMD activity might be depressed during pathogen infection, allowing the accumulation of alternate R gene transcripts which contain premature stop codons. When we examined the expression profiles in MOCK, AVR and VIR for the NMD related genes described in [31], we found that the observed patterns are consistent with weakened NMD in response to wounding and/or pathogen infection, with the greatest effect seen in AVR treated plants (see Files S23 and S24). In [32], the authors demonstrated that *Pst* DC3000-infected *Arabidopsis* mutants expressing loss of function mutations in genes of the NMD pathway showed *increased* disease resistance along with elevated transcription of critical defense response genes including *PR1*, and alternatively spliced *WRKY* transcripts. Interestingly, several of the intron retention events that we report have been previously described by other researchers and occur in genes critical to the *Arabidopsis* pathogen defense response. For example, Zhang and Gassmann [7] found that an intron retention event in the third intron of the *Arabidopsis* R gene, *RPS4*, is important during the defense response to *Pseudomonas*. The altered transcript is required for successful defense against *Pseudomonas*, suggesting that this transcript is not a target of NMD during plant defense. Although this intron retention is not currently annotated in TAIR10, our RNA-Seq results confirm the expression of this event. Similarly, Figure 9 displays a detected intron retention in the *SNC1* gene, which encodes another (TIR)-NB-LRR protein previously shown to be involved in the AvrRps4 modulated defense response to *Pst* [33], and subject to alternative splicing [34]. In this case, the intron retention event is not included in the standard TAIR10 annotation, but occurs in a B-list transcript.

The overall gene expression profile that we observe is consistent with previous studies and confirms and extends existing findings about genes likely to play an important role in the defense response to *Pseudomonas syringae*. For example, among a set of 3,705 genes exhibiting differential gene expression in a previous microarray study, the correlation between the microarray and RNA-Seq expression measurements was 0.81. Furthermore, several important pathogen response marker genes including *FRK1*, *PR1*, *PAD4* and *EDS1* were up-regulated in treated plants at the appropriate times during the course of infection, while lists of differentially expressed genes include many TIR-NB-LRR resistance genes, transcription factors and stress-response genes. Differentially expressed genes were highly enriched for a variety of relevant GO terms including ‘defense response’, ‘innate immune response’, ‘response to bacterium’, ‘programmed cell death’, ‘signal transduction’, ‘transcription factor activity’, and ‘transmembrane receptor activity’. Detailed lists of the genes that are differentially expressed between treatments at each time point are provided in the Supporting Information Files.

One of the main goals of our study was to also identify genes showing evidence for regulated alternative splicing associated with the plant's defense response. To achieve this goal, we assessed together with gene expression two additional types of differential expression: differential isoform expression (DEI) and differential isoform ratios (DIR). In the case of DEI, the goal is to identify each individual transcript that shows a statistically significant change in expression between treatments. Conceptually, differential gene expression and DEI are identical for genes that produce only one isoform, but they might differ for multi-isoform genes. In the case of DIR, the focus is on identifying multi-isoform genes where the relative ratios of the transcript isoforms generated by one gene change within each sample in response to treatment. Multi-isoform genes might show DEI, DIR, or both DEI and DIR. Consider, for example, a hypothetical 2-isoform gene co-expressing both isoforms. If the overall expression of the gene doubles, perhaps in response to a common transcription factor, the expression of each individual isoform is expected to increase as well, resulting in two DEI transcripts; however, the ratio of the two isoforms does not change. On the other hand, the same gene might show DIR, or DEI and DIR together, if the expression level of one isoform increases, while the other decreases.

Hence, in order to identify genes that are good candidates for regulated alternative splicing with possible biological relevance to the defense response, we focused on the set of genes with transcripts flagged as both DEI and DIR. File S11 contains a list of 105 genes having transcripts identified as both DEI and DIR. To further refine our candidates, we narrowed our focus to the subset of these genes where the observed splicing events occur in the vicinity of annotated pFAM domains, and where the isoform mixture percentage has changed by at least 10%. The resulting list of 29 genes is included in File S11 and summarized in Table 2. Interestingly, only 38% of these genes were identified as differentially expressed in tests for DEG. Furthermore, we also identified an additional set of 45 genes having large changes in isoform expression, but which lack annotated pFAM domains affected by splicing.

We have presented evidence suggesting that there are still a large number of previously unannotated alternatively spliced transcripts encoded in the *Arabidopsis* transcriptome. Some of these transcripts are likely to play an important role in the defense response against invasive pathogens. The set of high priority genes that we have identified in File S11 contains several extremely interesting candidates, including genes previously known to be alternatively spliced or expressed during the defense response. For example, in [7] the authors describe, in addition to *RPS4*, two genes that were differentially spliced in response to *Pst* DC3000 (AvrRps4) infection. One of these genes is At4g07410 (*POPCORN*), a transducin family WD-40 repeat protein. This gene has two annotated isoforms, At4g07410.1 and At4g07410.2, with At4g07410.2 lacking 1 out of 3 of the WD-40 domains contained in the first isoform. We observed that compared to mock-infected plants, the plants infected with the virulent strain of the pathogen displayed a 21% increase in the second isoform. Interestingly, the tests for DEG did not identify this gene as having a significant change in expression. In another example, we observed a 37% increase in the second isoform (At4g39270.2) of a leucine-rich repeat, transmembrane protein for AVR plants compared to VIR plants at 1 hpi. Once again, however, the gene was not identified as DEG. Compared to the first isoform, the At4g39270.2 splice variant contains an additional LRR domain. Although the function of this gene is unknown, it has previously been shown to be induced in response to flg22 and has been proposed to play a putative role in PTI defense [35]. Several other genes previously shown to play important roles in the defense response, including *SEN1* [36], *LURP1* [37], *JAR1* [38], and *EDR2* [39] were also identified as candidates for regulated alternative splicing, along with several additional interesting candidates, including two known splicing factor genes (At5g51300, *SR Protein 30*).

This paper also demonstrates that accurate quantification of alternative splicing using RNA-Seq is still a difficult problem. Current transcript assembly algorithms have high error rates, and incomplete annotations make it difficult to accurately assign a large portion of read pairs. This, together with positional biases inherent in RNA-Seq data sets, makes the accurate measurements of the relative expression levels of co-transcribed isoforms a challenging task [40], [41]. Nevertheless, most of these limitations are likely to be substantially mitigated contingent on continued progress in sequencing technology and statistical modeling. We hope that the extensive data set that we provide may serve as an important resource for generating hypotheses for continued biological research. Source code for the project is available at http://sourceforge.net/projects/iqowls.

## Methods

### Plant Material

All RNA-Seq experiments and subsequent validation experiments have been performed on four-week old soil-grown Columbia (Col-0) wild type plants, the reference genotype for *Arabidopsis*-related research. We carried out two independent inoculation experiments (biological replicates) for each treatment. Col-0 is susceptible to virulent *Pst* DC3000 but has a functional *RPS4* resistance gene effective against DC3000 expressing AvrRps4 [42]. Col-0 plants were inoculated with *Pst* DC3000 (empty vector) and *Pst* DC3000 (AvrRps4) at 107 cfu/ml, and mock-inoculated with buffer only (10 mM MgCl2) to control for non-specific effects resulting from injury or tissue flooding. Challenged leaf tissues were harvested from 10 plants per sample at 1, 6, and 12 hour post-inoculation (hpi). Total RNA was extracted using the Qiagen Plant RNeasy Mini kit (Qiagen, Valencia, CA-USA).

### RNA-Seq Alignment

#### IQ.OWLS and EdgeR

We first aligned read pairs to the TAIR 10 transcriptome using Bowtie version 0.12.5 [11], with the following parameters: -a (report all valid alignments); –solexa1.3-quals (FASTQ quality scores are ASCII chars equal to Phred quality plus 64); -n 2 (alignments are allowed no more than 2 mismatches for each read in a pair); –trim5 4 –trim3 5 (trim the first 4 and last 5 bases before aligning); −l 66 (read length = 66); −I 75 (min fragment length = 75); −X 5500 (max fragment length = 5500); –fr (upstream read goes in fwd direction; downstream paired read goes in reverse direction). For runs 2 and 3, which had 75 nucleotide reads, we trimmed 4 nt from the beginning and 5 nt from the end; for run 4, which had 76 nucleotide reads, we trimmed 5 nt from the beginning and 5 nt from the end; for run 5 which had 100 nucleotide reads, we trimmed 4 nt from beginning and 30 nt from the end.

All reads that did not result in unspliced bowtie alignments to an existing TAIR 10 transcript were subsequently aligned to the TAIR 10 genome using the TopHat spliced alignment tool version 1.2.0 [12]. The following TopHat parameters settings were used: –solexa1.3-quals (FASTQ quality scores are ASCII chars equal to Phred quality plus 64); -I 3000 (maximum intron length in nucleotides); -i 15 (minimum intron length in nucleotides); –mate-std-dev 135 (standard deviation for inner distance between mate pairs); -G TAIR10_GFF3_genes.gff (use TAIR 10 gene annotations).

Subsequently, all alignments were processed with a custom Java program and uploaded into a mySQL database. The Java program parses the alignments and counts how many mismatches occur in each pair of reads. It also identifies read pairs which map to more than one gene. Given a gene with *n* unique AS isoforms, that gene's RNA-Seq read pairs can be partitioned into 2*^n^*-1 categories 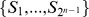 according to the subset of the gene's isoforms each read pair is compatible with. More precisely, for each alignment pair having 0 mismatches, and mapping unambiguously to a single gene locus, the program assigns the corresponding fragment to the matching subset of compatible transcripts 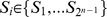. Fragments that occur within 300 nucleotides of a TAIR10 gene locus, but which do not correspond to any known transcript of this locus are assigned to an additional subset 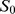, see Methods S1.

#### Cufflinks

The various Cufflinks applications are designed to work directly on spliced alignments to the genome. Reconciliation with known gene models then occurs as an optional downstream step. We used the TopHat program, version 1.2.0, to align the set of all read pairs to the TAIR 10 genome, using the following command line options: –solexa1.3-quals,-I 3000, -i15, -r125, –mate-std-dev 150, -F 0, -G TAIR10_GFF3_genes.gff.

### Distribution of Fragment Start and Fragment Size

The empirical distributions for fragment start position and fragment size were estimated separately for each of the 18 RNA samples. See Files S21 and S22. First, we took a random sample of 10 read pairs aligned to each single isoform gene in TAIR 10; in case fewer than 10 read pairs were available for a gene, all available alignments were used. To estimate the distributions for fragment start positions, the resulting random samples were divided into bins according to the length of the target transcript, with bin boundaries at every 100 nucleotides. Within each bin, we fit a smoothing spline in R to estimate the empirical distribution of read start position. This procedure was applied separately for genes on the forward and reverse strands. Fragment size distributions were estimated in a similar manner, using a kernel density procedure to estimate the distribution of the differences between the start positions of paired reads.

### Differential Expression of Genes and Transcripts

We used three different approaches to identify genes and transcripts that were differentially expressed between treatments.

#### EdgeR Classic

The first method, described in the documentation by its authors as EdgeR ‘classic’, is implemented in the EdgeR Bioconductor package in R [15]–[17]. To identify differentially expressed genes, we first counted the number of read pairs aligned uniquely to each TAIR 10 gene with 0 mismatches. Within each time point, we then used EdgeR to compare the resulting read counts from the two biological replicates for each pair of treatments. Differential expression p-values were computed using the EdgeR “ExactTest” method using moderated, tagwise dispersion [16]. We discarded genes where the total read count was less than 10 in each treatment and computed adjusted p-values for the remaining genes using the Benjamini-Hochberg method for false discovery correction [43]. Genes that had an FDR-adjusted p-value less than or equal to 0.10 were identified as differentially expressed in a given pair of treatments.

We employed a similar approach to identify differentially expressed transcripts among the multi-isoform genes in TAIR 10. In this case, we considered only read pairs that mapped uniquely to each transcript (e.g. S_1_ and S_2_ reads for the two isofom case as defined in the Methods section). Once again, we discarded transcripts where the total read count was less than 10 in both treatments and identified differentially expressed transcripts having FDR-adjusted p-values less than or equal to 0.10.

#### EdgeR GLM

This method makes use of recently developed functionality within the same EdgeR package described above. The EdgeR general linear models framework allowed us to specify a design matrix that estimates the effect of run number (batch) as a nuisance parameter. In contrast to the EdgeR classic method, this approach fits a model for all samples simultaneously. In addition, this method employs alternative procedures for estimating the dispersion (the functions ‘estimateGLMTrendedDisp’ and ‘estimateGLMTagwiseDisp’) and for model fitting (‘glmFit’). As in the case of the EdgeR classic method, the response variable was the number of read pairs uniquely mapping to each gene (for differential gene tests) or isoform (for differential transcripts tests). After fitting the model, we defined contrasts between experimental treatments and tested for significant expression differences using a likelihood ratio test (‘glmLRT’). We discarded genes and transcripts where the total read count was less than 10 in each treatment and computed adjusted p-values for the remaining genes using Benjamini-Hochberg method for false discovery correction. Genes and transcripts that had an FDR-adjusted p-value less than or equal to 0.10 were identified as differentially expressed in a given pair of treatments.

#### Cufflinks

We experimented with several Cufflinks parameters settings. We achieved the best results using the following parameters. Read pairs were first aligned to the TAIR 10 genome using TopHat version 1.2.0, as described above. Subsequently, the cuffdiff program (version 1.2.1) was used to identify differentially expressed genes and isoforms. The arguments to the program consisted of the alignment files for the two replicates of each treatment along with the TAIR 10 gene models. Genes and transcripts that were reported as having a status of ‘OK’ and a q value less than or equal to 0.10 were considered to be differentially expressed.

### Isoform Abundance Estimation

In addition to determining which transcripts and genes were differentially expressed between treatments, we were also interested in quantifying the relative ratios of each isoform for the multi-isoform genes in each sample. For this purpose we examined two different approaches.

#### IQ.OWLS

The first method, which we call “IQ.OWLS” (Isoform Quantification Obtained by Weighted Least Squares), is described in [44]. This model, along with extensions that accommodate paired end reads and biological replicates are reviewed in Methods S1.

#### Cufflinks

We also used the Cufflinks program to estimate isoform abundances for the annotated TAIR 10 isoforms. After aligning reads to the TAIR 10 genome, we ran Cufflinks version 1.2.1 with the following command line options: -G TAIR10_GFF3_genes.gff, -I 3000, -i 15. For each multi-isoform gene, transcript percentages were computed by calculating relative frequencies from the FPKM values in the resulting isoforms.fpkm_tracking output files.

### Detection of Novel Genes, Transcripts and Splicing Events

#### Splicing Events

The basic procedure for identifying novel splicing events consists of a) identifying reads that are inconsistent with known TAIR 10 gene models, but which overlap known genes, b) searching among those reads for evidence for a set of standard alternative splicing patterns such as intron retention, exon skipping, etc., and c) identifying such events that are expressed above a threshold level. The procedures used to detect novel UTR extensions, intron retention, exon skipping, cryptic introns, cryptic exons and alternative 3 and 5′ splice sites are detailed in Methods S1.

#### Novel Genes and Transcripts

We used the Cufflinks program to identify novel genes and transcripts. First, we aligned the RNA-Seq read pairs to the TAIR 10 genome as described above. Subsequently, for each of the resulting alignments, we ran Cufflinks version 1.2.1 using the default parameters. In particular, no reference GFF file was provided. The resulting assemblies were then consolidated and reconciled with known TAIR 10 gene models using the cuffmerge program.

### GO Term Analysis

We used agriGO, a web-based GO Analysis Toolkit and Database for Agricultural Community (http://bioinfo.cau.edu.cn/agriGO/, [21]) to identify enriched GO terms in our gene lists. In all cases we employed the following parameter settings: hypergeometric test, with Yekutieli FDR adjustment, 0.10 significance level, 5 minimum mapping entries and ‘Complete GO’ ontology type. For analysis of differential alternative splicing of known TAIR 10 transcripts, we used the set of 5,885 multi-isoform genes in TAIR 10 as the background reference. For analysis of novel intron retention, novel cassette exon, and novel alternative 3′/5′ splice site events we used the set of 22,523, multi-exon genes in TAIR 10 as the background reference. Otherwise, we used the suggested background, “*Arabidopsis* genemodel (TAIR)”.

### qPCR Validation

For each sample, total RNA was quantified with a NanoDrop ND-1000 (Nanodrop, Delaware, USA) and 600 ng total RNA was used for first-strand cDNA synthesis (GoScript Reverse Transcription system, Promega) using a mixture of random hexamer primers and oligo dT. Quantitative real-time PCR (qPCR) was performed on a Statagene Mx-3000P QPCR system (Agilent Technologies, CA, USA). PCR parameters were as recommended by the supplier. In short, preincubation was performed at 95°C for 10 min, followed by 40 amplification cycles consisting of a 15s incubation at 95°C, a 30s incubation at 55°C and a 30s incubation at 72°C. 1ul first-strand cDNA per reaction was used for the quantitative PCR analysis. All reactions were measured in triplicate using PerfeCTa SYBR Green SuperMix, Low ROX (Quanta Biosciences). Primers (Invitrogen) for qPCR analysis were designed using PRIMER3 [45]. Transcript-specific primers were designed to specifically anneal to exon-exon junctions corresponding to each splice form. PP2A (At1g69960) was used as the reference gene. Transcript List and primer sequences are provided in Methods S1.

## Supporting Information

File S1 **Data and Alignment Statistics.**
(XLSX)

File S2 **QCSpreadsheets.**
(ZIP)

File S3 **FPKM.**
(XLSX)

File S4 **DEG 1 hpi.**
(XLSX)

File S5 **DEG 6 hpi.**
(XLSX)

File S6 **DEG 12 hpi.**
(XLSX)

File S7 **DEI and DIR 1 hpi.**
(XLSX)

File S8 **DEI and DIR 6 hpi.**
(XLSX)

File S9 **DEI and DIR 12 hpi.**
(XLSX)

File S10 **GoTerms.**
(PDF)

File S11 **All DEI and DIR Genes.**
(XLSX)

File S12 **qPCR Validation of Isoform Quantification.**
(XLSX)

File S13 **Summary_Transcript Extensions.**
(XLSX)

File S14 **Summary_SpliceJunctions.**
(XLSX)

File S15 **Novel IR Events.**
(XLSX)

File S16 **Novel CI Events.**
(XLSX)

File S17 **Novel DA Events.**
(XLSX)

File S18 **Novel CE Events.**
(XLSX)

File S19 **Novel KE Events.**
(XLSX)

File S20 **Cufflinks Gene Models.**
(XLSX)

File S21 **StartPos Distribution.**
(XLSX)

File S22 **PairOffset_Distribution.**
(XLSX)

File S23 **STEM Profiles for NMD Genes.**
(DOCX)

File S24 **STEM Profiles for NMD Genes – Details.**
(XLSX)

Methods S1 **Supplementary Methods.**
(DOCX)
