# Supplementary material for: High-Throughput RNA Sequencing of Pseudomonas-Infected Arabidopsis Reveals Hidden Transcriptome Complexity and Novel Splice Variants
Source: PLoS One. 2013 Oct 1;8(10):e74183. doi: 10.1371/journal.pone.0074183 (PMC3788074; doi:10.1371/journal.pone.0074183)
Supplement: File S23 — STEM Profiles for NMD Genes. (DOCX) [file pone.0074183.s023.docx]

# Procedure

- Used a list of NMD related genes from (Rayson et al., 2012):

  Table S1 = Genes that are co-ordinately up-regulated in NMD mutant Arabidopsis (common NMD genes)
  Table S2 = Genes that are co-ordinately down-regulated in NMD mutant Arabidopsis
- After cross-referencing these gene lists with genes in TAIR 10, obtained a list of 203 genes up-regulated in NMD mutants ("NMD_mut up") and 131 genes down-regulated in NMD mutants ("NMD_mut down")
  - In general, NMD_mut up genes, which increase when NMD is knocked out, are candidates for direct regulation by NMD
  - NMD_mut down genes, which decrease when NMD is knocked out may be indirectly regulated by NMD. For example, NMD may down-regulate a repressor of these genes. When NMD is knocked out, the repressor increases, and the NMD_mut down gene decreases.
- For each gene in the combined NMD_mut up/down list, obtained the total IQ.OWLS FPKM expression level (summing over the two replicates) for: MOCK 1hpi, AVR 1hpi, VIR 1hpi, MOCK 6hpi, AVR 6hpi, VIR 6hpi, MOCK 12hpi, AVR 12hpi, VIR 12hpi.
- Used this data to load three separate FPKM expression time series for MOCK, AVR and VIR into the STEM (Ernst & Bar-Joseph, 2006) clustering tool (with 15 max profiles, log expression values, otherwise default settings)
- STEM assigned each gene to at most one of the 15 candidate profiles in each treatment (MOCK, AVR, VIR), and identified enriched profiles.

# Hypothesis

NMD plays a role in defense response; possibly during the course of infection, NMD is turned off or mitigated in order to alter the regulation of defense related transcripts.

If this hypothesis is true, we would expect to see:

- NMD_mut up genes should trend upward in expression during the course of the infection.
- This effect might be more pronounced in AVR and VIR compared to MOCK.
- NMD_mut down genes should trend downward during the course of the infection.
- This effect might be more pronounced in AVR and VIR compared to MOCK

# Results

- Significant profiles for the three treatments, using the combined NMD_mut up/down FPKM lists are shown in color below:

MOCK


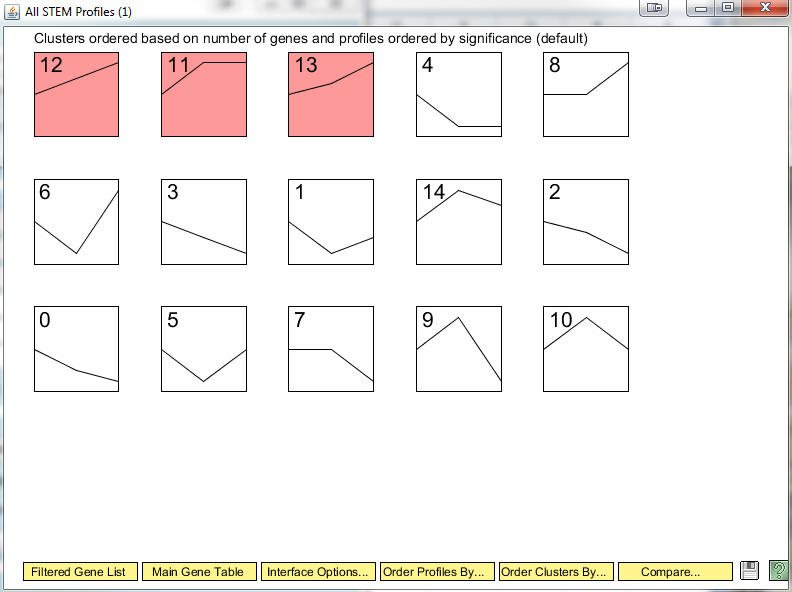


AVR


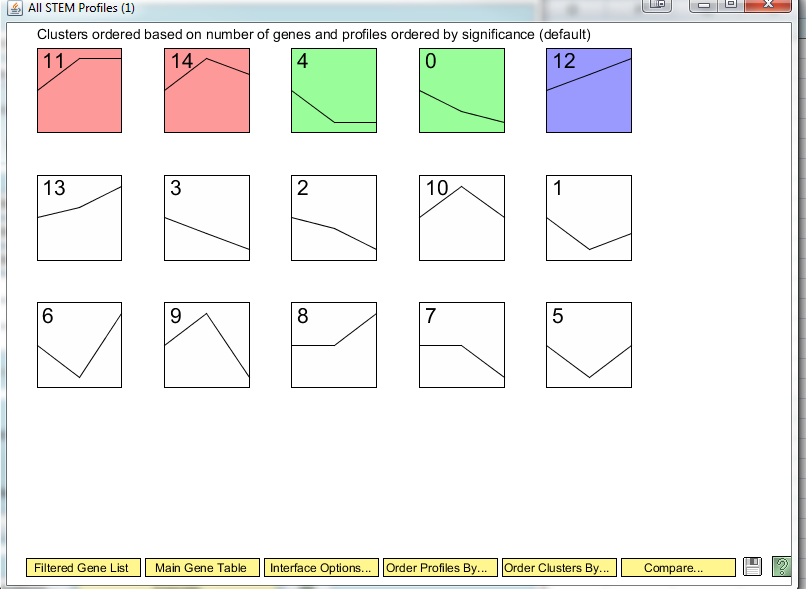


VIR


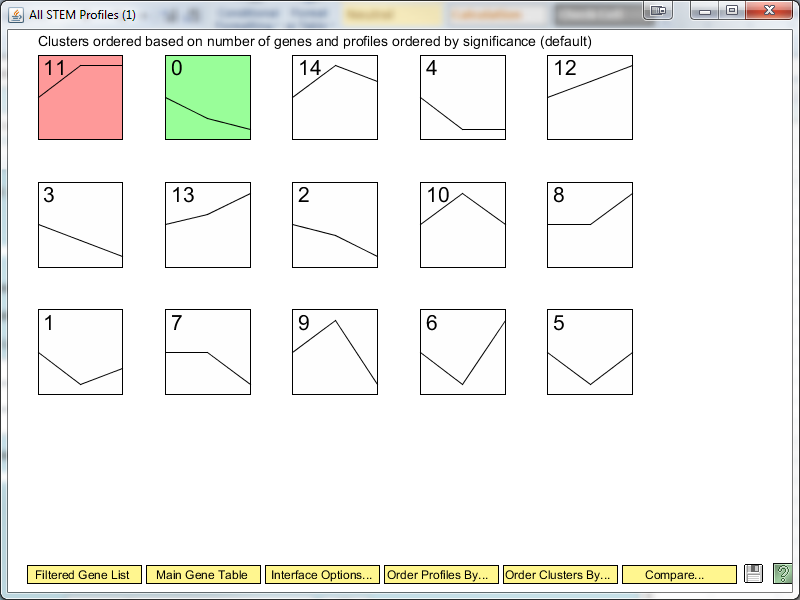


These tables summarize gene counts from NMD_mut up and NMD_mut down genes in each profile. (See also "S24 - STEM Profiles for NMD Genes-Details.xlsx."):

| NMD mutant up | | | Down Profiles | | | | |  | Up Profiles | | | | | |
| --- | --- | --- | --- | --- | --- | --- | --- | --- | --- | --- | --- | --- | --- | --- |
| **Profile** | **4** | **3** | | **2** | **0** | **7** | **Total** |  | **Profile** | **12** | **11** | **13** | **8** | **Total** |
| **Mock** | 19 | 5 | | 2 | 2 | 3 | 31 |  | **Mock** | 14 | 21 | 10 | 20 | 65 |
| **Avr** | 12 | 3 | | 2 | 3 | 4 | 24 |  | **Avr** | 19 | 62 | 6 | 2 | 89 |
| **Vir** | 6 | 8 | | 3 | 9 | 5 | 31 |  | **Vir** | 11 | 50 | 6 | 3 | 70 |
|  | | | | | | | | | | | | | | |
| NMD mutant down | | | Down Profiles | | | | |  | Up Profiles | | | | | |
| **Profile** | **4** | **3** | | **2** | **0** | **7** | **Total** |  | **Profile** | **12** | **11** | **13** | **8** | **Total** |
| **Mock** | 9 | 1 | | 1 | 1 | 1 | 13 |  | **Mock** | 19 | 15 | 8 | 7 | 49 |
| **Avr** | 11 | 5 | | 4 | 10 | 5 | 35 |  | **Avr** | 2 | 20 | 1 | 0 | 23 |
| **Vir** | 7 | 0 | | 7 | 10 | 8 | 32 |  | **Vir** | 4 | 24 | 1 | 0 | 29 |

- In the following:
  - profiles 0,2,3,4 and 7 are "down-trending" profiles
  - profiles 8, 11,12, and 13 are "up-trending" profiles
  - profiles 1,5,6,9,10, and 14 are "other" profiles

### Observations:

**For NMD_mut up genes:**

- there are more genes assigned to up-trending profiles than are assigned to down-trending profiles:
  - the overall ratio is 224 up / 86 down = 2.6
  - for Mock the ratio is 65 up / 31 down = 2.09
  - for Avr the ratio is 89 up / 24 down = 3.87
  - for Vir the ratio is 70 up / 31 down = 2.26
- In Avr and Vir there are a higher percentage of genes in profiles 11 and 12 compared to Mock; in Mock there is a higher percentage of genes in profiles 8 and 13 compared to Avr and Vir
- these observations are consistent with the hypothesis: NMD_mut up genes are more likely to show an up-trend during the course of the infection, and this tendency is reduced (slightly) and delayed in Mock

**For NMD_mut down genes:**

- in AVR and VIR, there are slightly more genes assigned to down profiles than up profiles, but in MOCK there are more than 3 times as many more genes assigned to up profiles than down profiles.

up / down ratios:

MOCK 49 up /13 down: 3.77

AVR 23 up /35 down: 0.66

VIR 29 up /32 down: 0.91

- Hence, NMD_mut down genes tend to decrease during the course of the infection, but only for AVR and VIR. This is consistent with the hypothesis that NMD is decreased for AVR and VIR during the infection.

**For NMD_mut up AND down genes:**

- All but one of the significant STEM profiles was either an 'up' or 'down' profile. The only significant "other" profile was profile 14 in AVR.
- Down profiles were only found to be significant in AVR and VIR.

# Conclusions

- Observed expression profiles are not inconsistent with those expected if NMD were weakened in response to wounding and/or disease progression
- The effect appears to be smaller and/or delayed in Mock treatment compared to Avr and Vir
- The effect may be slightly larger in Avr compared to Vir.

# References

Ernst, J., & Bar-Joseph, Z. (2006). STEM: a tool for the analysis of short time series gene expression data. *BMC bioinformatics*, *7*, 191. Retrieved from http://www.pubmedcentral.nih.gov/articlerender.fcgi?artid=1456994&tool=pmcentrez&rendertype=abstract

Rayson, S., Arciga-Reyes, L., Wootton, L., De Torres Zabala, M., Truman, W., Graham, N., Grant, M., et al. (2012). A role for nonsense-mediated mRNA decay in plants: pathogen responses are induced in Arabidopsis thaliana NMD mutants. *PloS one*, *7*(2), e31917. doi:10.1371/journal.pone.0031917
