# Supplementary material for: High-Throughput RNA Sequencing of Pseudomonas-Infected Arabidopsis Reveals Hidden Transcriptome Complexity and Novel Splice Variants
Source: PLoS One. 2013 Oct 1;8(10):e74183. doi: 10.1371/journal.pone.0074183 (PMC3788074; doi:10.1371/journal.pone.0074183)
Supplement: Methods S1 — Supplementary Methods. (DOCX) [file pone.0074183.s025.docx]

Supplementary Methods

High-Throughput RNA Sequencing of Pseudomonas-Infected Arabidopsis Reveals Hidden Transcriptome Complexity and Novel Splice Variants

# 1. Distribution of Read Start and Fragment Size

In most RNA-Seq experiments, reads are not sampled uniformly along the length of a given transcript. Instead, the shape of the true sampling distribution depends on experiment-specific factors including transcript length, read length, fragmentation protocol, and sequencing technology [1]. We estimated, for each of the 18 RNA-Seq samples, the empirical distribution of fragment position as a function of transcript length (Figure 15a, b). For each sample, the read distributions were estimated separately for genes on the forward and backward chromosome strands, and the resulting density plots indicate a partial mRNA degradation in 5' to 3' direction [2]. The distributions for each sample are available in the supplementary file "S21 - StartPos Distribution.xlsx."

Similarly, we used our data to estimate the empirical distribution of fragment lengths in each of the 18 samples. The implied fragment size was measured in terms of the difference between read start positions in each pair (rightmost read start – leftmost read start). The mean pair offset differed somewhat between samples, but in general, the offset distributions were unimodal, and bell shaped (Figure 15c). The distributions for each sample are available in the supplementary file "S22 - PairOffset_Distribution.xlsx."

# 2. Isoform Quantification with IQ.OWLS

The following describes the isoform quantification method "IQ.OWLS" (Isoform Quantification Obtained by Weighted Least Squares). This material contains a review of the method as previously described in [3,4], along with various extensions to accommodate paired-read data, multiple biological replicates, and bootstrap confidence intervals.

## 2.1 Model Overview and Extension to Paired Reads

Given a set of *n* unique AS isoforms for a gene, *g*, it is always possible to partition RNA-Seq reads aligning to *g* into 2*n* categories according to the subset of known isoforms with which each read is compatible. For example, consider two AS isoforms, T1 and T2:

T1: AAAAAAA UUUUUUUUUU CCCCCCCCCC

T2: AAAAAAA ---------- CCCCCCCCCC

In this example, transcript T1 contains a cassette exon containing only “U” nucleotides. Transcript T2 skips this exon. Unpaired reads aligned to this gene can be classified into 4 mutually-exclusive subsets:

- *Subset S0*: Reads that are not compatible with either transcript T1 or T2.
- *Subset S1*: Reads that are only compatible with transcript T1, e.g. UUUUUUU.
- *Subset S2*: Reads that are only compatible with transcript T2, e.g. AAACCCC.
- *Subset S3*: Reads that are compatible with both T1 and T2, e.g. AAAAAAA.

For the purposes of quantification we will ignore S0 reads and only consider reads that align to at least one of the known isoforms. However, a large number of S0 reads may indicate that the transcript catalog is incomplete. Note that for paired reads, both reads in each pair must be considered together as a unit. For example, if the first read in a pair contains only A's and the second read in the pair contains only C's, the pair belongs to subset S3; if the first read contains only A's and the second read contains only U's, then the pair belongs to subset S1. In the following, we will use the term "fragment" to denote either a single unpaired read, or a pair of reads sequenced from opposite ends of the same physical cDNA fragment. Let:

Pr(Si) denote the probability that one of the gene’s fragments aligns to subset Si

Pr(Tj) denote the probability that one of the gene’s fragments aligns to transcript Tj

denote the percentage of the gene’s fragments expressed as isoform Tj

Given the subsets introduced above, the following equation describes the probability that an individual fragment aligns to subset Si:

We assume that , the probability an individual fragment aligns to a particular transcript T*j*, is dependent on the (unknown) frequency, , of that transcript in the transcript mixture. We also assume that a given isoform is sampled with probability proportional to its known length. Similarly, $\text{Pr}\left(\text{S}_{i}|\text{T}_{j}\right)$, the probability that an individual fragment maps to subset S*i*, given that the fragment aligns to transcript Tj, can be worked out using the known transcript sequence and estimates of the distributions for fragment length and fragment start position (for details, see the section “Constructing the Design Matrix” below). Let:

*Yi* denote the number of fragments compatible with subset Si

*R* denote the total number of fragments aligned to the gene

*pij* = $\text{Pr}\left(\text{S}_{i}|\text{T}_{j}\right)$

$\beta_{j}$ =

Under this model:

For the example shown above, we can express this linear model in matrix form as follows:

$$\left[\begin{matrix} Rp_{11} & 0 \\ 0 & Rp_{22} \\ Rp_{31} & Rp_{32} \end{matrix}\right]\left[\begin{matrix} \beta_{1} \\ \beta_{2} \end{matrix}\right]=\left[\begin{matrix} Y_{1} \\ Y_{2} \\ Y_{3} \end{matrix}\right]$$

Because *Rp*21 and *Rp*12 will always be zero, the rank of this matrix is 2, and both 1 and 2 are estimable. Although, in general, the number of rows (2*n* – 1) grows exponentially with the number of possible transcripts, it is possible to either combine or ignore uninteresting categories. In fact, a full rank design matrix can often be constructed by considering only the *n* subsets consisting of fragments that map to a single isoform. In Arabidopsis the majority of the multi-isoform genes (4,318 out of 5,885) have only two annotated isoforms in TAIR 10, but a minority of transcripts have between 3 and 10 isoforms. Using our method we were able to quantify AS in all of these transcripts.

## 2.2 Distribution of Fragment Start Position and Fragment Length

Many methods for estimating isoform abundance assume a uniform sampling distribution for fragments along the targeted transcripts. However, it is widely acknowledged that the true distribution for fragment position deviates substantially from uniformity, and varies with the fragmentation protocol and sequencing technology [1]. Consequently, accurate methods for isoform quantification should incorporate this information. We use a kernel density approach to estimate fragment start distributions given the observed empirical distributions observed for well-annotated single isoform transcripts.

For paired reads, we also incorporate knowledge about the distribution of fragment length. We encode fragment length information in terms of a "pair offset" between the two reads in each pair. The distribution of fragment length in each sample is estimated from the set of expressed single isoform genes.

## 2.3 Constructing the Design Matrix

Let *h*( *k*, *o* | *L* ) denote the bivariate probability mass function describing the probability that a fragment has start position *k* and pair offset *o*, given that this fragment aligns to a transcript of length *L*. Assuming that the distribution of fragment sizes, *j*( *o* ), and fragment start positions *g*( *k* | *L* ) are independent,

*h*( *k*, *o* | *L* ) = *j*( *o* ) *g*( *k* | *L* )

We compute$\text{Pr}\left(\text{S}_{i}|\text{T}_{j}\right) \forall i\in\left\{1\ldots2^{n}-1\right\}$for a particular transcript Tj using the procedure detailed in BOX1.

## 2.4 Combining Design Matrices for Two Treatments plus Replicates

Here, we are interested in pairwise comparisons of treatments, each of which consists of two biological replicates. A straightforward extension to the design described above allows us to estimate these parameters simultaneously. Let $\beta_{iA}$ denote the ith parameter for treatment A, and let $\beta_{iB}$denote the ith parameter for treatment B. The following linear system illustrates, for the two isoform case, how one can simply stack design matrices and read counts to enable joint estimation:

$$\left[\begin{matrix} X_{A1} & 0 \\ X_{A2} & 0 \\ 0 & X_{B1} \\ 0 & X_{B2} \end{matrix}\right]\left[\begin{matrix} \beta_{1A} \\ \beta_{2A} \\ \beta_{1B} \\ \beta_{2B} \end{matrix}\right]=\left[\begin{matrix} Y_{A1} \\ Y_{A2} \\ Y_{B1} \\ Y_{B2} \end{matrix}\right]$$

where

- $X_{A1}$represents the 3x2 design matrix computed as described above for treatment A, replicate 1
- $X_{A2}$, $X_{B1}$, and $X_{B2}$ are the 3x2 design matrices corresponding to treatment A, replicate 2 and treatment B replicates 1 and 2 respectively.
- $Y_{A1}$ is the length 3 vector of subset counts observed in treatment A, replicate 1
- $Y_{A2}$, $Y_{B1}$, and $Y_{B2}$ are the length 3 vector of subset counts observed in treatment A, replicate 2 and treatment B replicates 1 and 2 respectively.

## 2.5 Distributional Model For Subset Counts

We used the EdgeR BioConductor R package [5–7] to model the distribution of subset counts. For each pairwise comparison, we prepared a table of subset counts for all multi-isoform genes from the samples A1, A2, B1 and B2. We then used EdgeR to compute maximum likelihood estimates for the mean value of each subset count in each treatment, along with the corresponding moderated tagwise dispersions. These resulting estimates allow us estimate the variance of each subset count and to perform bootstrap sampling of subset counts under EdgeR's negative binomial model.

## 2.6 Estimation of and

Given the design matrix construction method described above, the design matrix will always be full column rank, so $\hat{\beta}$ will always be fully estimable. Under the Aitken linear model with a known covariance matrix, the parameter estimate obtained using weighted least squares is the best linear unbiased estimator (BLUE) for Beta [8]. We used the EdgeR negative binomial variances for subset counts to construct a diagonal covariance matrix for weighted least squares estimation.

In cases where a resulting $\hat{\beta}$ is not a valid probability, we truncate the estimate at 0 or 1. In addition, we ensure that the total probability is one by dividing each $\hat{\beta}_{iA}$ by $\sum_{i} \hat{\beta}_{iA}$ and each $\hat{\beta}_{iB}$ by $\sum_{i} \hat{\beta}_{iB}$.

Under the commonly employed assumption that a given isoform is sampled with probability proportional to its length, the probability that a given fragment maps to a transcript isoform can be expressed as follows:

where *Lj* is the length of transcript *j* in bases, and the '.' in$\beta_{j\cdot}$ and is a place holder for either treatment A or B. Given our estimates for the *j*’s, the known lengths for each transcript, and the fact that , the are uniquely determined and can be obtained by computing the unique solution to a set of linear equations.

## 2.7 Multireads

An important consideration when dealing with RNA-Seq reads is that many fragments can actually align to more than one gene. Our method discards fragments that align to more than one gene, after making an appropriate adjustment to the design matrix. When computing the probabilities as described in BOX1,for a given transcript each fragment is checked against all other genes in the target dataset. If the fragment does not map to any other genes, the corresponding probability $\text{Pr}\left(\text{S}_{i}|\text{T}_{j}\right)$ is updated, otherwise the fragment is skipped without any update. This procedure can be performed efficiently after pre-computing a *k*-mers hash table.

## 2.8 Bootstrap Confidence Intervals and Differential AS

In order to estimate the variability of our estimates we computed parametric bootstrap confidence intervals for each and for each difference. Using EdgeR's estimates for the negative binomial parameters for each subset, we randomly generated 2000 replicate bootstrap samples for each subset count. For each of these samples, the simulated subset counts were used to estimate each using the method described above; after the simulation, the 95% bootstrap confidence interval was computed for each parameter. Differential AS was identified by finding genes with at least 75 total aligned read pairs in treatments A and B, and which have bootstrap confidence intervals for that do not include zero.

## Box 1

Assume fixed read length for each fragment end = *m.*

Initialize $\Pr\left(\text{S}_{\text{i}}\text{|}T_{\text{j}}\right)=0,\text{ }i \in\left\{1\ldots2^{n}-1\right\}$.

For each possible fragment start site, *k*, along transcript T­j :

For each possible pair offset, *o*, for a fragment starting at position *k* :

Test the read pair against a *m-*mer hash of multireads: if both reads in the pair map to two or more different genes, we skip this fragment.

Otherwise:

Determine the sequence for the first read in the pair, with start position *k*, and end position *k*+*m*-1.

Determine the compatibility of this sequence with each of the *n* transcripts $\text{T}_{\text{x}}, x\in\left\{1\ldots n\right\}$.

Determine the sequence for the second read in the pair, with start position *k*+*o*, and end position *k*+*o*+*m*-1.

Determine the compatibility of this sequence with each of the *n* transcripts $\text{T}_{\text{x}}, x\in\left\{1\ldots n\right\}$.

The intersection of the two sets of transcript compatibilities determines which subset, Si, the read pair belongs to.

For the appropriate Subset, update the conditional probability as follows:
$\begin{matrix} \Pr\left(\text{S}_{\text{i}}\text{|T}_{\text{j}}\right)=\Pr\left(\text{S}_{\text{i}}\text{|T}_{\text{j}}\right)+h\left(k,o\text{|}L_{j}\right) \\ =\Pr\left(\text{S}_{\text{i}}\text{|T}_{j}\right)+j\left(o\right)g(k|L_{j}) \end{matrix}$

# 3. Identification of Novel Splicing Events

Novel splicing events were identified by mining the set of read pairs that were not consistent with any TAIR 10 transcript, but which had valid spliced alignments that overlapped with TAIR 10 genes (S0 reads). A given read pair was assigned to a particular gene if that read pair overlapped within 300 nucleotides of the gene's annotated coordinates, and if the read did not also overlap one or more other genes. In the following, multiple read pairs having the same length and start position were only counted once, in order to eliminate potential PCR artifacts.

### 3.1 Splice junctions

We first compiled the set of all TAIR 10 splice junctions to assess the number of known and novel splice junctions covered by RNA-Seq read pairs. For each of the 128,271 splice junctions in TAIR 10, we counted the number of distinct supporting S0 and non-S0 read pairs. We then examined the S0 read pairs and identified novel splice junctions not reported in TAIR 10. The results are recorded in the file "S14 -Summary_SpliceJunctions.xlsx."

### 3.2 Transcript extension

Among the S0 read pairs, we counted the number of read pairs that overlapped within 300 nucleotides of each TAIR 10 gene's annotated range, and which did not also overlap with another gene. These read pairs were identified as supporting an extension to the annotated transcript(s) for that gene. In the majority of cases, this is expected to represent an extension (or addition) of an untranslated region at either the 3' or 5' end of an existing transcript. However, in some cases, transcript extension may instead correspond to an alteration of the annotated translated reading frame of the existing gene model. The results are recorded in the file "S13 - Summary_Transcript Extensions.xlsx."

### 3.3 Novel Intron Retention

The following procedure was used to identify novel intron retention (IR) events:

- For each multi-exon gene from TAIR 10 (22,523 out of 33,602 genes), the introns in each transcript were examined sequentially. RNA-Seq reads that cover these introns, but which do not otherwise map to a known gene model (S0 reads) may provide evidence of a novel intron retention event.
- Any intron that overlaps an exon from a different transcript from the same gene was immediately excluded from consideration, since some reads that cover these introns might actually originate from the overlapping transcripts rather than from an intron retention event.
- Similarly, any intron that overlaps an exon from another gene was also excluded.
- Given the remaining introns (about 90% ) we used the RNA-Seq data to compute
  1. the minimum S0 read coverage along the intron
  2. the mean S0 read coverage along the intron
- High values for a) and b) increase our confidence in the novel IR. Given the user defined threshold values t1 and t2, novel intron retention events can therefore identified according to the following criteria:
  - the minimum S0 coverage (a) must be ≥ t­1
  - the mean S0 coverage (b) must be ≥ t2
- Here, we used t1 (minimum S0 coverage) equal to 1 and t2 (mean S0 coverage) equal to 2. Events are recorded in the file "S15 - Novel IR Events.xlsx", along with values for a) and b) as defined above.

### 3.4 Cryptic Introns

Novel cryptic intron events were identified using the following procedure:

- For each transcript from TAIR 10, the exons were examined sequentially. We examined reads that have spliced alignments to the genome, but which do not map to a known gene model (S0 reads). S0 reads that contain an apparent splice junction that is completely contained inside a known exon may provide evidence for a novel cryptic intron.
- Exons that overlap introns in other gene models, either from the same gene or a different gene were excluded from consideration. This is a conservative approach that eliminates many instances where an apparent cryptic intron might actually reflect an alternative splice pattern between known exons, such as alternative 5' and 3' splice sites.
- For each remaining exon, we identified the set of all unique S0 splice junctions completely contained inside the exon. Two detected splice junctions were considered to be different from one another if they have different 5' and/or 3' splice sites. For each unique splice junction we recorded the number of distinct S0 reads supporting the junction.
- Splice junction reads that were confirmed by at least t4 = 2 distinct reads were accepted as candidates for intron retention events, the results are recorded in the file "S16 - Novel CI Events.xlsx."

### 3.5 Cassette Exons

Novel cassette exons were identified using the following procedure:

- For each gene from TAIR 10, we first identified all possible sets of three consecutive exons occurring within the same transcript for that gene. A novel cassette exon event can then be detected by observing evidence for a splice junction linking the 3' end of the first exon in the set to the 5' boundary of the third exon (thereby skipping the second exon).
- After recording in advance all possible splice junctions that would support novel cassette exons in TAIR 10 genes according to the above definition, we next examined the set of S0 reads that have spliced alignments to the genome, but which do not map to a known gene model. S0 reads that are consistent with the precomputed set of cassette exon junctions provide evidence for novel cassette exon events.
- Given these criteria, splice junction reads that were confirmed by at least t5 = 2 distinct reads were accepted as candidates for novel cassette exon events, the results are summarized in the file "S18 - Candidate Novel CE Events.xlsx."

### 3.6 Cryptic Exons

Novel cryptic exons were identified using the following procedure:

- For each multi-exon gene from TAIR 10, the introns in each transcript were examined sequentially. Any intron that overlaps an exon from a different transcript of the same/different gene was immediately discarded, since some reads that cover these introns might actually originate from the overlapping transcripts rather than from a novel cryptic exon event.
- We identified introns containing cryptic exon events in the remaining introns according to the following criteria:

1. the intron must contain evidence for exactly one novel splice junction (called "junction 1," possibly supported by more than one S0 read) with a left end that coincides with the left end of the intron, and the right end of the junction located inside of the intron.
2. the intron must contain evidence for exactly one novel splice junction (called "junction 2," possibly supported by more than one S0 read) with a right end that coincides with the right end of the intron, and the left end of the junction located inside of the intron.
3. the right end of the junction in a) must be to the left of the left end of the junction in b).

- For introns having such events, we computed:
- the total number of unique reads (with different start sites) supporting junction 1
- the total number of unique reads (with different start sites) supporting junction 2
- the minimum S0 read coverage of the presumed novel exon
- the mean S0 read coverage of the presumed novel exon
- Given the threshold values t6, t7, t8, and t9, novel cryptic exon events can be identified according to the following criteria:
  - the number of reads supporting junction 1 (a) must be ≥ t­6
  - the number of reads supporting junction 2 (b) must be ≥ t­7
  - the minimum S0 coverage (c) must be ≥ t8
  - the mean S0 coverage (d) must be ≥ t9
- We used t6 equal to 2, t7 equal to 2, t8 equal to 1, and t9 equal to 2; the results are recorded in the file "S19 - Novel KE Events.xlsx."

### 3.7 Alternative 3' or 5' Splice Site

Novel alternative 3' and 5' splice sites were identified using the following procedure:

- An alternative 3' splice site is detected when the 5' end of a detected splice junction matches a known splice junction, but the 3' side does not. As a result, the exon on the 3' side of the junction is extended or shortened. Alternative 5' splice sites are detected in an analogous way.
- We examined all multi-exon genes in TAIR 10, and identified spliced S0 reads that matched the above pattern. The 3' end of a novel alternative 3' splice junction read was required to have no match to any gene model, and to occur at a location subsequent to the 5' border of the previous exon in the corresponding gene. In the case of novel 5' splice junctions, the procedure proceeds analogously.
- For each identified event we recorded:
  - the number of unique reads (with different start sites) supporting the junction
  - in the cases where the splicing event extends an exon into an intronic region, the minimum read count and the mean read count covering this region
- Given threshold values t10, t11, and t12, novel alternative 3' and 5' splicing events are identified according to the following criteria:
  - the number of reads supporting junction 1 (a) must be ≥ t­10
  - the minimum S0 coverage (b) must be ≥ t11
  - the mean S0 coverage (c) must be ≥ t12
- We used t10 equal to 2, t11 equal to 1, and t11 equal to 2; the results are recorded in the file "S17 - Novel DA Events.xlsx."

# 4. qPCR Primers

| **Transcript** | **Left Primer** | **Right Primer** |
| --- | --- | --- |
| at1g01550.1 | CCTGGAAATTTCTGTCCACAA | TGGACGAGCCATTCTGATAA |
| at1g01550.2 | CCGACTCTTATTTCTTATCTTC | TTGTGGACGAGCCATTC |
| at1g51620.1 | TGTCCAATGCACAAGATCCA | GTCTGCATCACCTGCAAAGA |
| at1g51620.2 | ATTTGCTGTGATGCAGACGA | CCTCCTTTGCCATGAACTCT |
| at3g23280.1 | TGAAGAAACGAAGGGTCTGC | GGCTGTGGAATCCCTTTACA |
| at3g23280.2 | TAACTCCACAAAAACACGTCT | CCCATGGCTAGTCCATCTT |
| at4g38810.1 | AACCCTCTAGCTGTCTTATCCGTA | TGTCCGACAACACTTCTTTGA |
| at4g38810.2 | CCTTTTCTCGTGCTTCCATC | ACTTGTTGCTGTTGCTGGTG |
| at5g17760.1 | ATAATCCGCTCGTCTCCA | TTAGTGTAACAAGACTGACTCTGA |
| at5g17760.2 | TCAATGGTCCCTGAGACTCG | TGTGATGCGTGACTCTGATCT |
| at5g41750.1 | GTTCCCTTATGCGACTCCAA | ACAATCCACCGATGTTCCAT |
| at5g41750.2 | AGAATATGAGCCTGAAGCTGTT | CGACCTAAGTTGCATGTTTTG |
| at1g04990.1 | CCAATGATCTCTCTCTGCCC | AGTCAAAAACAGAAGAGAAACCTGTGA |
| at1g04990.2 | CCAATGATCTCTCTCTGCCC | CAAACTCTATTTTTCAACATCACTG |
| at1g07350.1 | TGTTGAGAAGGCAAGACGTC | GCGGGGAGAGTAGCTAGGAG |
| at1g07350.2 | TCACCAAATAGCAAACGTTG | CAGCCCCAAGTACTTTCCTG |
| at1g09140.1 | GCTCTGTCTCAAGGTCCCAAT | CCAGTGGCCAGTTTTCATTT |
| at1g09140.2 | TTTTTACGGTCTGGTCTGGTCT | TAGTCCCGGGAGCTTAAATG |
| at1g19660.1 | AAATTTTCGAGTGAAGATGAGGTC | TTTTTGGTCTGGTTACCCCA |
| at1g19660.2 | TGGGGTGATGTTGGATACTAAAA | AACACTAGGGCAAACAACCG |
| at1g55210.1 | TCAAGAACGGTGATGCTACTG | ACACACACCATTAAACCAAATG |
| at1g55210.2 | TCAAGAACGGTGATGCTACTG | ATTATCAAATCTTTCAAACCACCAA |
| at1g56520.1 | AATTCGAGTCTTACAAAGCTTACGA | TGTTCCGTTTGGAACGTGTA |
| at1g56520.2 | TTGTGTTAGTCATTGGCGACTC | ATAATGCGAGCGTAACCACC |
| at1g64990.1 | GTTTTTGCAGATCTGAGGTTTTT | AACCTGCCCATCCTAAGAGA |
| at1g64990.2 | CCAAATAAACCCTAACCAAAAATAA | CCATAAACCTGCCCATCCTA |
| at2g46610.1 | TATGAAGTCTGGTTATGCTTTTGTG | TTCACCCTGAAAGTCCTTGG |
| at2g46610.2 | AACGTTTTCTTGCACTTGTACTCC | ACACAACCCCAGTCATGGTT |
| at4g02600.1 | ACATTCCAGTGTTGGTACATAAAAG | TCCTCCGTGACCCATTTTAC |
| at4g02600.2 | GAATCTGTCTCACTGATTTCTTCATCT | GTGAATTCAAGCGACATCCC |
| at4g04620.1 | GGTTTCGAGTTTTGTTTCTCTTAATC | CCACAATCACGGGAACTCTT |
| at4g04620.2 | TCGTCTTTTGGATACTAACCGAATC | CCACAATCACGGGAACTCTT |
| at4g29210.1 | GATCACCAAGGATGGTGAGTTC | GCCATTGATCGTGGTGAAGT |
| at4g29210.2 | ATTTTTTTATCGCAGGATGGTGA | TTGATCGTGGTGAAGTTCTCA |
| at4g39270.1 | AAGAAAGTGTTCCTGGGTCTGC | GATTCCGAGTTTCCCTGTGA |
| at4g39270.2 | CAAGCTTGCTAATTTCTTAACTATTCT | GCTCCAGAAGTATCTTGCCG |
| at5g04830.1 | GTGAAAATGAAACAGCGATGG | TTGGCTTTTGCACTAGACCC |
| at5g04830.2 | AATCCGAATTCAGCGATGGA | AAATCGTTGGCTTTTGCACT |
| at5g63120.1 | TGTTTCCTCTGGTATTAGGGAAAAAAG | CAGGCTGCTCAAAAACCAAT |
| at5g63120.2 | AGGGGACTTGATGTGAAGGAC | TCTTGACAAGCTCTCTCGCA |
| at1g69960.1 (PP2A) | TGCCCCAAACTATTGCTACC | GGAGTCTTGCGAGTGGTTTC |

# 5. References

1. Wang Z, Gerstein M, Snyder M (2009) RNA-Seq: a revolutionary tool for transcriptomics. Nature reviews Genetics 10: 57–63. Available: http://www.pubmedcentral.nih.gov/articlerender.fcgi?artid=2949280&tool=pmcentrez&rendertype=abstract. Accessed 31 March 2011.

2. Muhlrad D, Decker CJ, Parker R (1994) Deadenylation of the unstable mRNA encoded by the yeast MFA2 gene leads to decapping followed by 5’-->3' digestion of the transcript. Genes & Development 8: 855–866. Available: http://genesdev.cshlp.org/cgi/content/abstract/8/7/855. Accessed 21 September 2011.

3. Howard B, Veronese P, Heber S (2011) Improved RNA-Seq Read Partitions in Linear Models for Isoform Quantification (accepted for publication). IEEE International Conference on Bioinformatics & Biomedicine (BIBM).

4. Howard BE, Heber S (2010) Towards reliable isoform quantification using RNA-SEQ data. BMC bioinformatics 11 Suppl 3: S6. Available: http://www.pubmedcentral.nih.gov/articlerender.fcgi?artid=2863065&tool=pmcentrez&rendertype=abstract. Accessed 17 May 2011.

5. Robinson MD, Smyth GK (2007) Moderated statistical tests for assessing differences in tag abundance. Bioinformatics (Oxford, England) 23: 2881–2887. Available: http://bioinformatics.oxfordjournals.org/cgi/content/abstract/23/21/2881. Accessed 16 June 2011.

6. Robinson MD, Smyth GK (2008) Small-sample estimation of negative binomial dispersion, with applications to SAGE data. Biostatistics (Oxford, England) 9: 321–332. Available: http://www.ncbi.nlm.nih.gov/pubmed/17728317. Accessed 1 November 2010.

7. Robinson MD, McCarthy DJ, Smyth GK (2010) edgeR: a Bioconductor package for differential expression analysis of digital gene expression data. Bioinformatics (Oxford, England) 26: 139–140. Available: http://www.pubmedcentral.nih.gov/articlerender.fcgi?artid=2796818&tool=pmcentrez&rendertype=abstract. Accessed 11 June 2011.

8. Monahan JF (2008) A Primer on Linear Models (Chapman & Hall/CRC Texts in Statistical Science). Chapman and Hall/CRC. Available: http://www.amazon.com/Primer-Linear-Chapman-Statistical-Science/dp/1420062018. Accessed 8 October 2012.
