# Supplementary material for: High-Throughput RNA Sequencing of Pseudomonas-Infected Arabidopsis Reveals Hidden Transcriptome Complexity and Novel Splice Variants
Source: PLoS One. 2013 Oct 1;8(10):e74183. doi: 10.1371/journal.pone.0074183 (PMC3788074; doi:10.1371/journal.pone.0074183)
Supplement: File S10 — GoTerms. (PDF) [file pone.0074183.s010.pdf]

DEG 6hpi - Biological Process

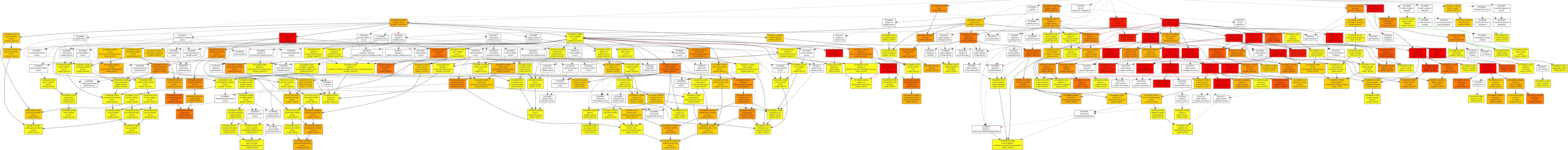

DEG 6hpi - Cellular Component

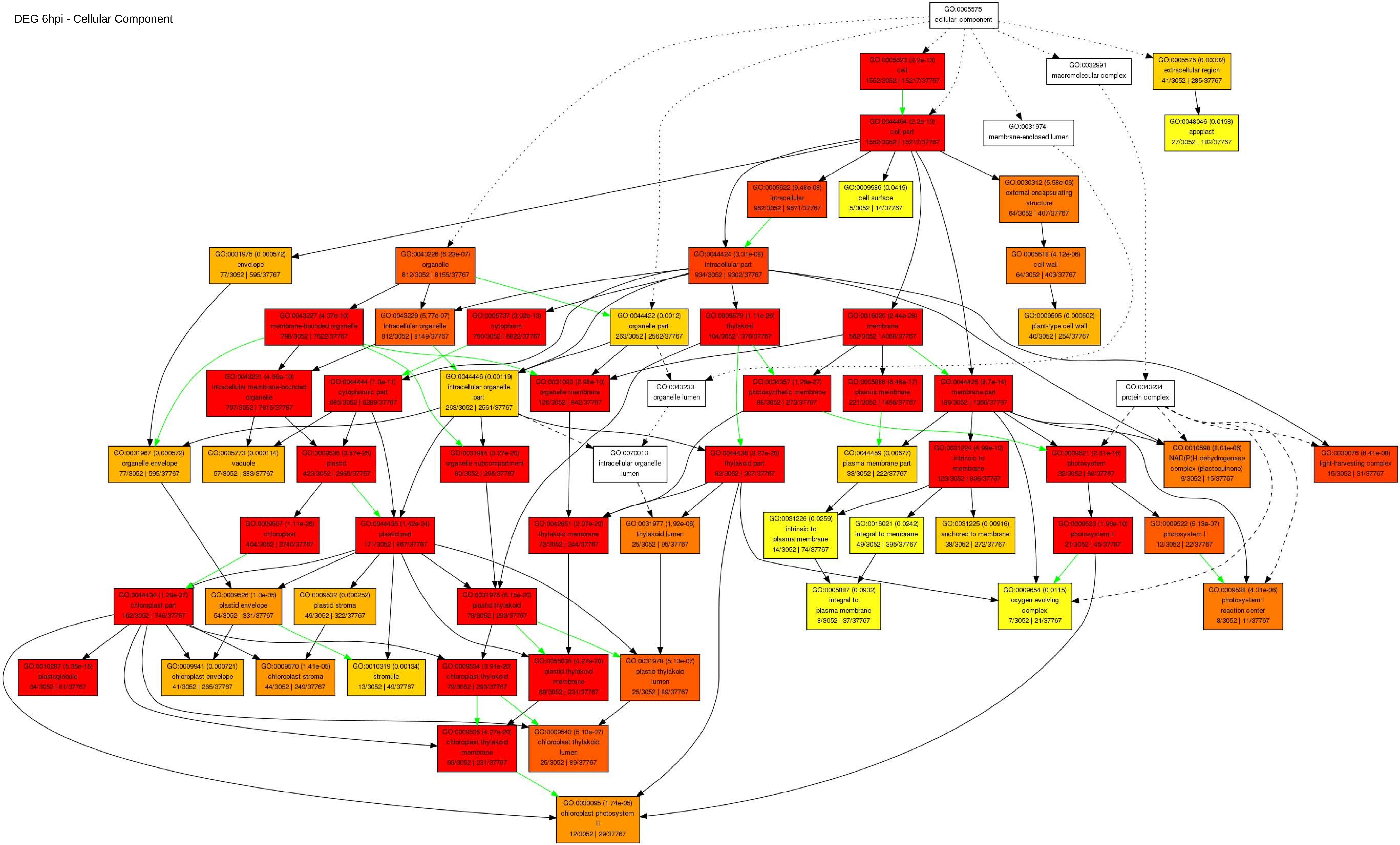

DEG 6hpi - Molecular Function

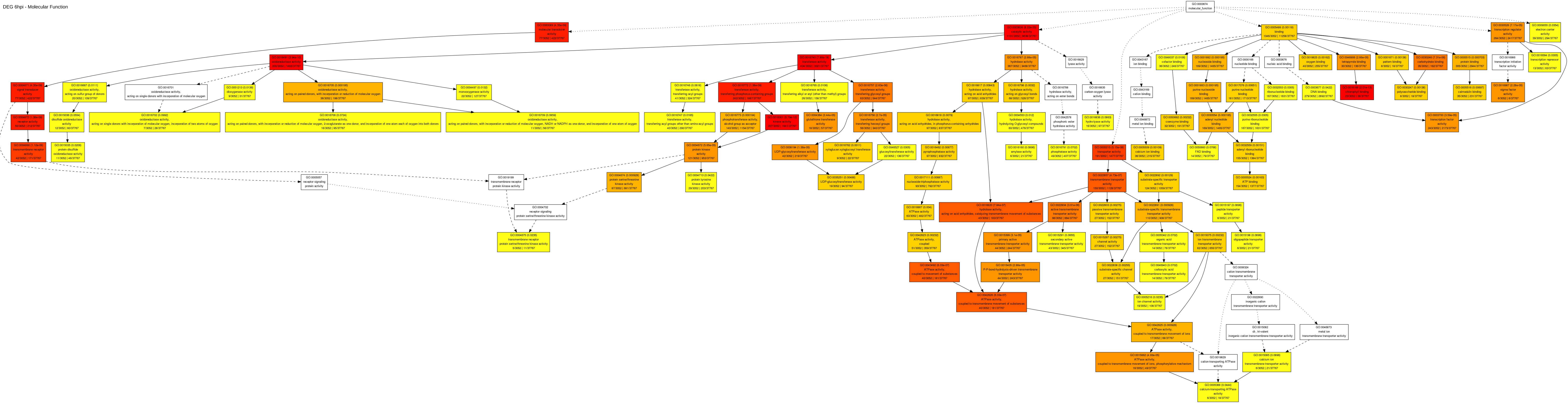

Novel Intron Retentions - Biological Process

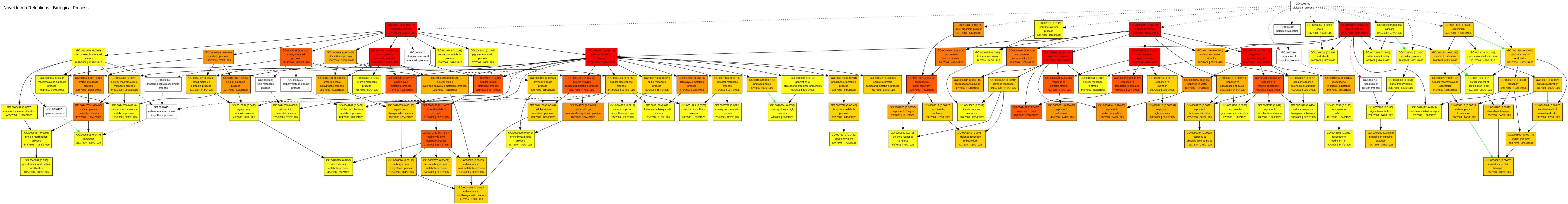

Novel Intron Retentions - Cellular Component

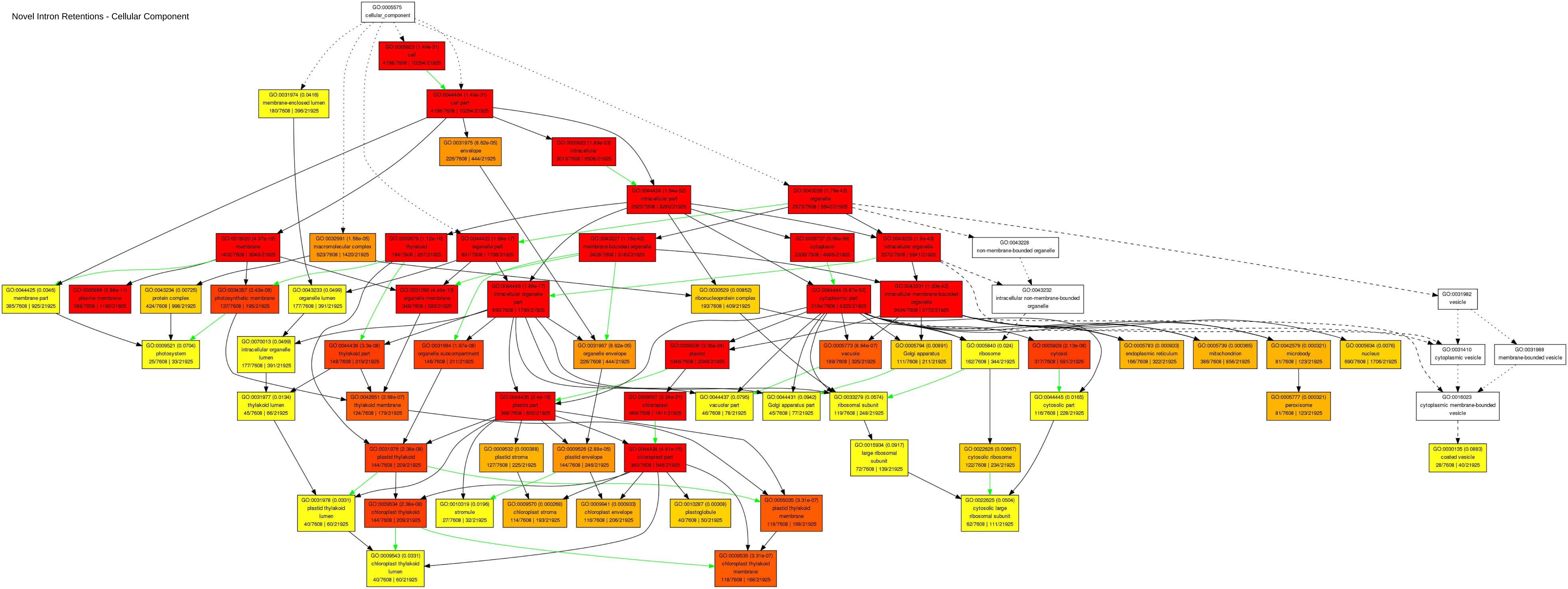

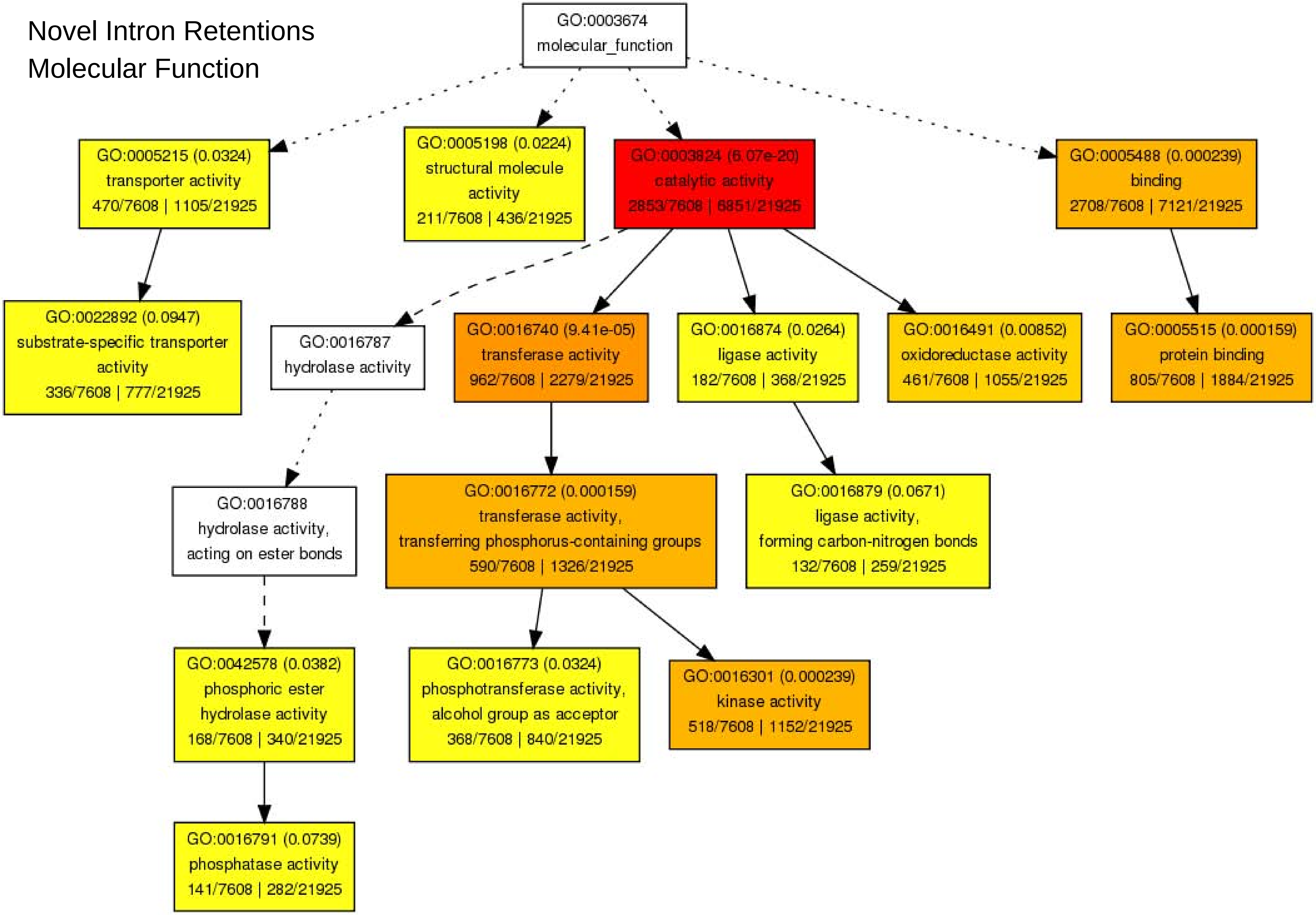
Novel Intron Retentions
Molecular Function
GO:0003674
molecular_function
GO:0005215 (0.0324)
transporter activity
470/7608 | 1105/21925
GO:0005198 (0.0224)
structural molecule activity
211/7608 | 436/21925
GO:0003824 (6.07e-20)
catalytic activity
2853/7608 | 6851/21925
GO:0005488 (0.000239)
binding
2708/7608 | 7121/21925
GO:0022892 (0.0947)
substrate-specific transporter activity
336/7608 | 777/21925
GO:0016787
hydrolase activity
GO:0016740 (9.41e-05)
transferase activity
962/7608 | 2279/21925
GO:0016874 (0.0264)
ligase activity
182/7608 | 368/21925
GO:0016491 (0.00852)
oxidoreductase activity
461/7608 | 1055/21925
GO:0005515 (0.000159)
protein binding
805/7608 | 1884/21925
GO:0016788
hydrolase activity, acting on ester bonds
GO:0016772 (0.000159)
transferase activity, transferring phosphorus-containing groups
590/7608 | 1326/21925
GO:0016879 (0.0671)
ligase activity, forming carbon-nitrogen bonds
132/7608 | 259/21925
GO:0042578 (0.0382)
phosphoric ester hydrolase activity
168/7608 | 340/21925
GO:0016773 (0.0324)
phosphotransferase activity, alcohol group as acceptor
368/7608 | 840/21925
GO:0016301 (0.000239)
kinase activity
518/7608 | 1152/21925
GO:0016791 (0.0739)
phosphatase activity
141/7608 | 282/21925

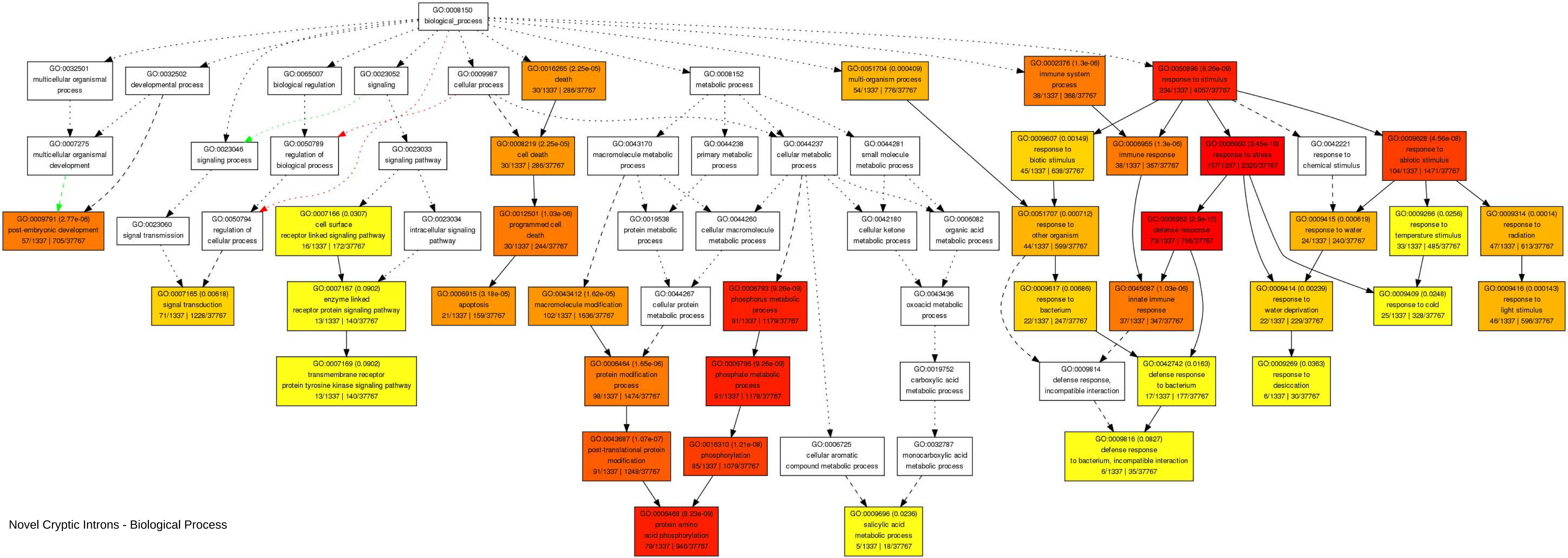

Novel Cryptic Introns - Biological Process

Novel Cryptic Introns - Cellular Component

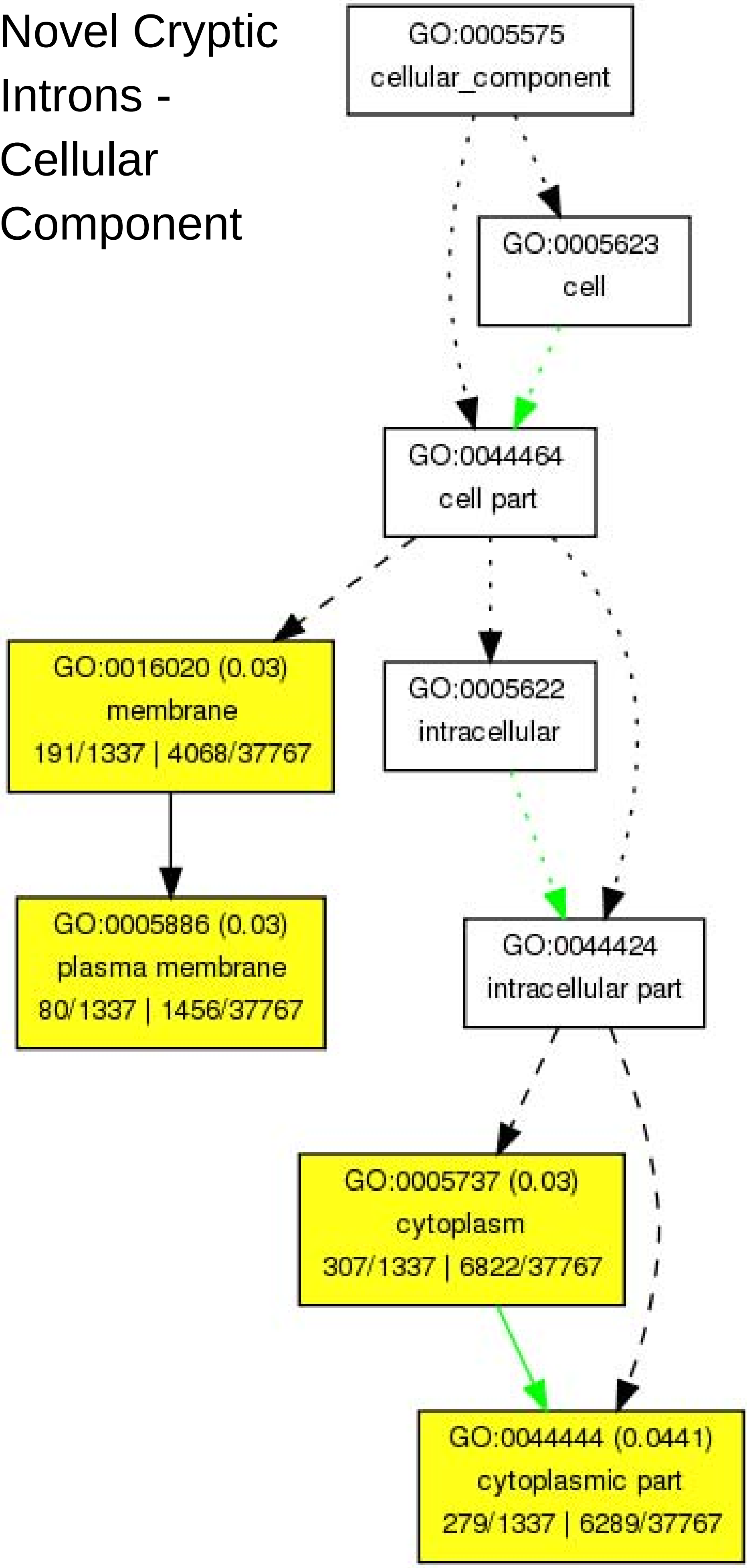

Novel Cryptic Introns - Molecular Function

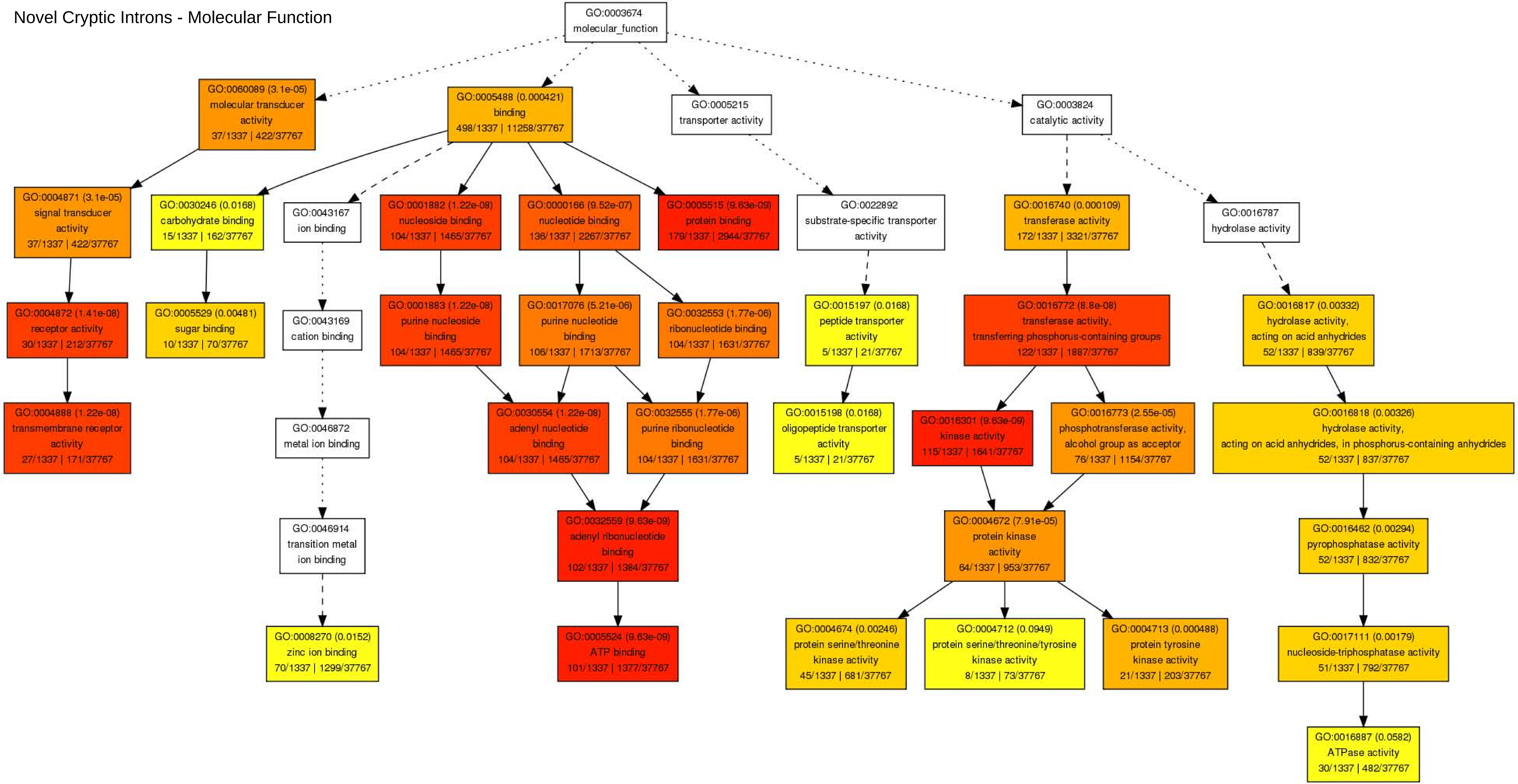

Novel Cassette Exons
Biological Process

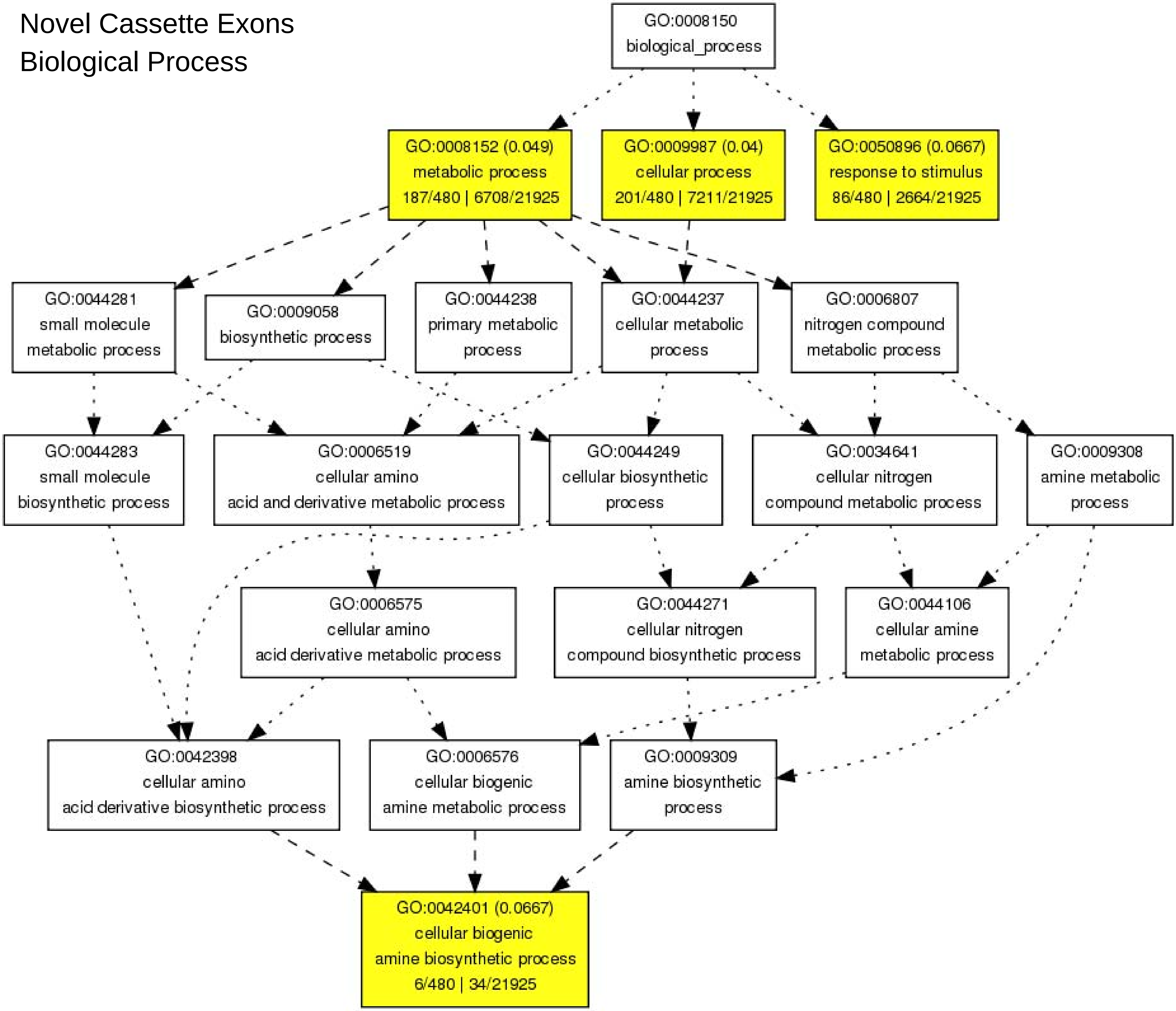

Novel Cassette Exons
Cellular Component

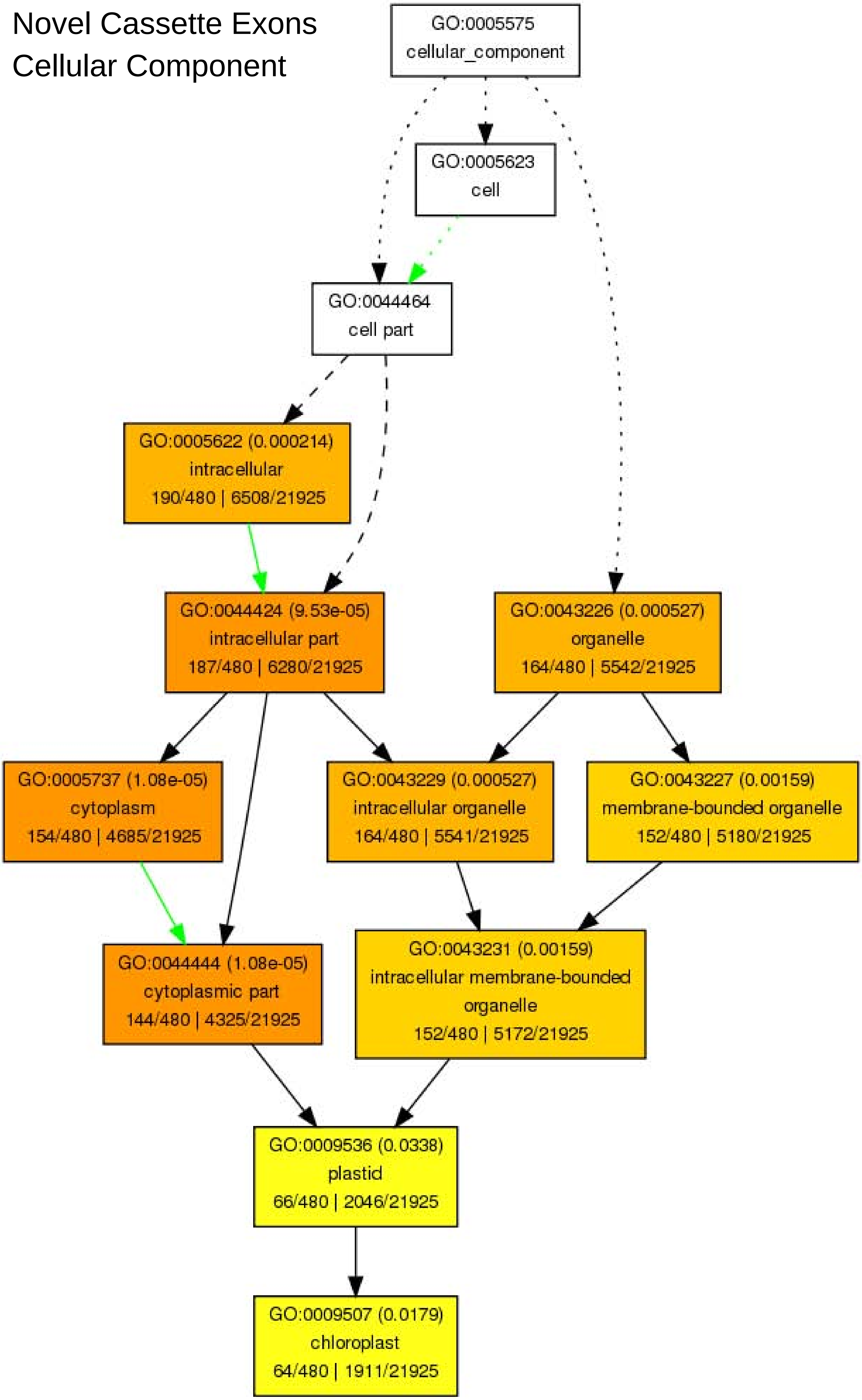

Novel Cryptic Exons
Biological Process

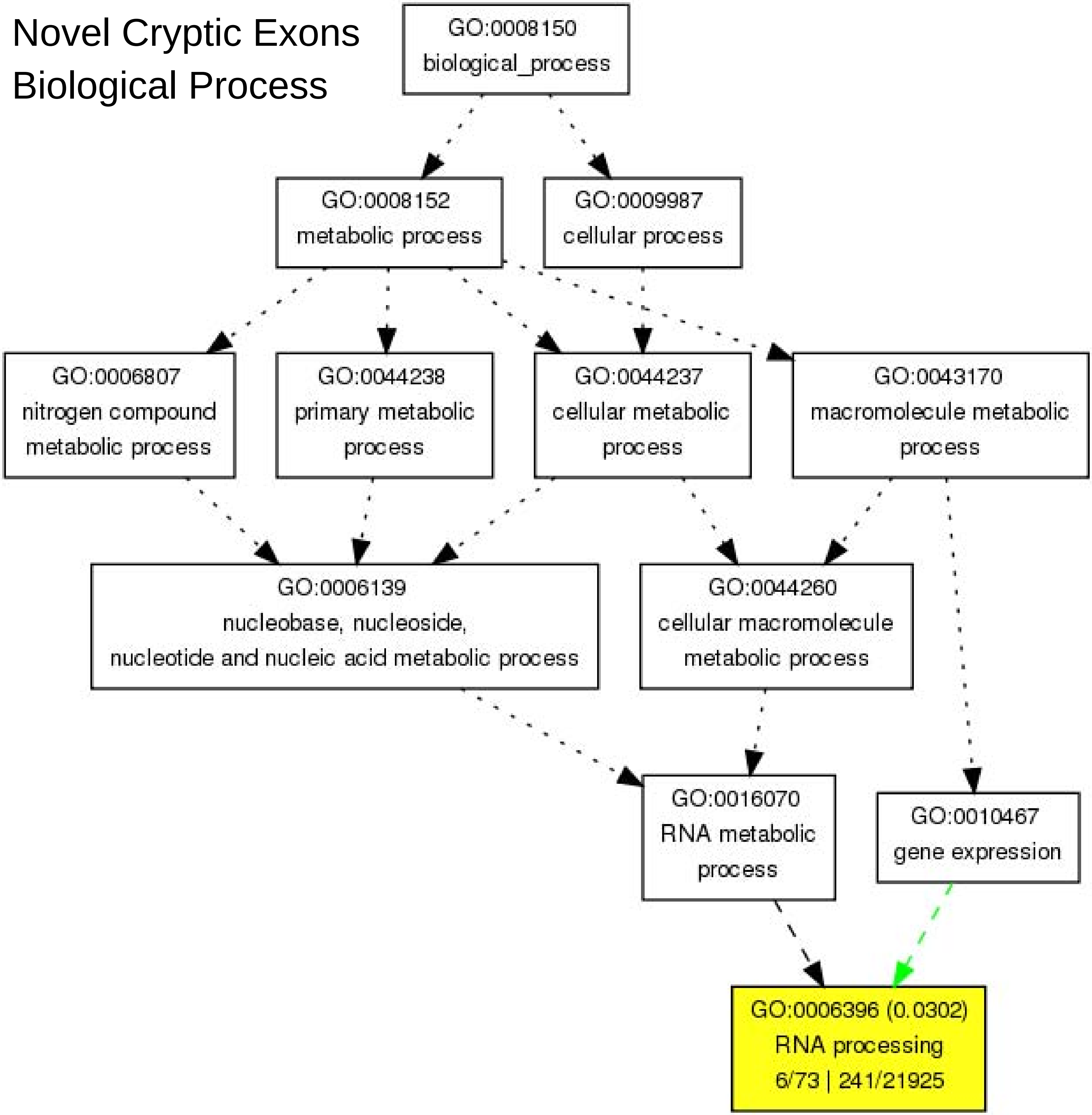

Novel Cryptic Exons Molecular Function

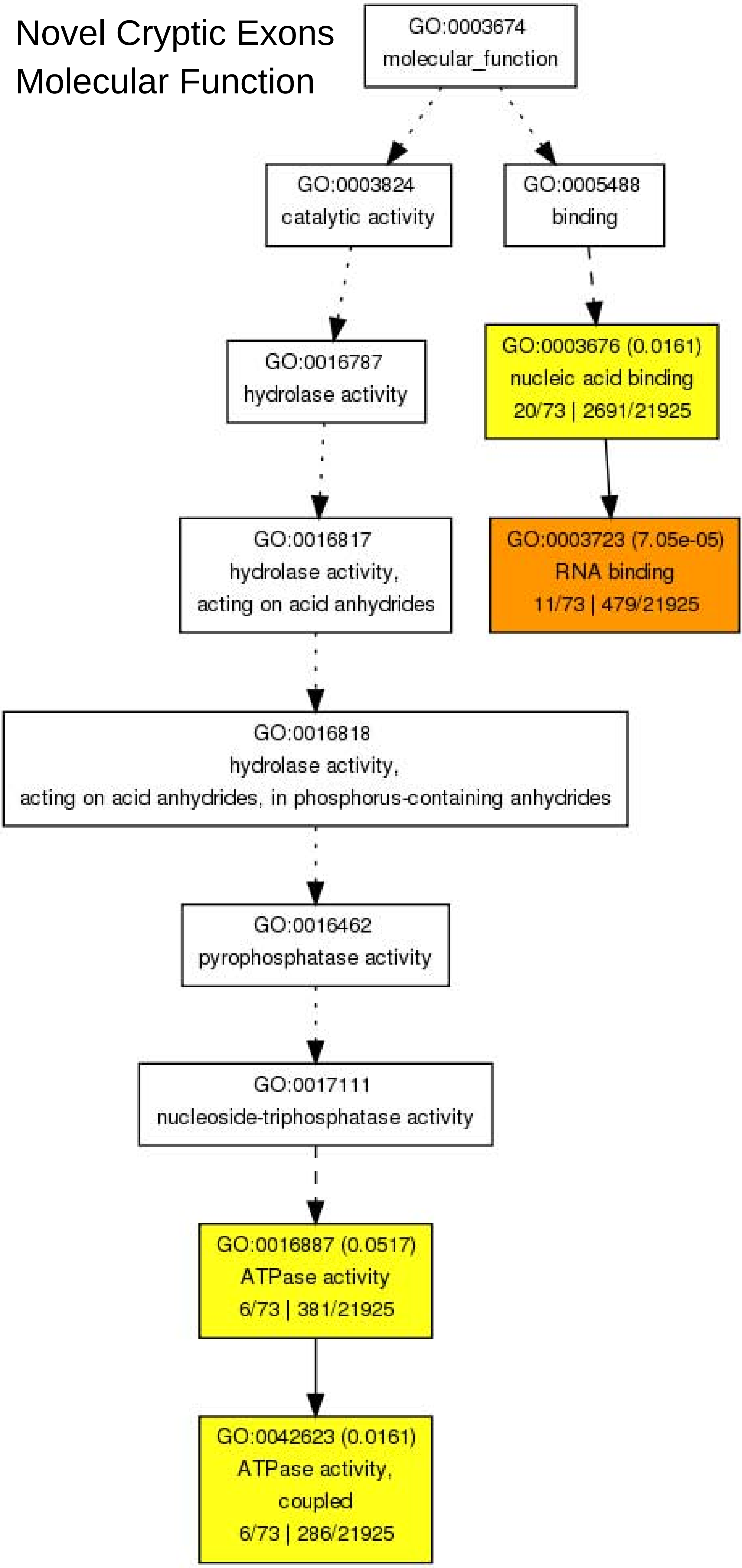

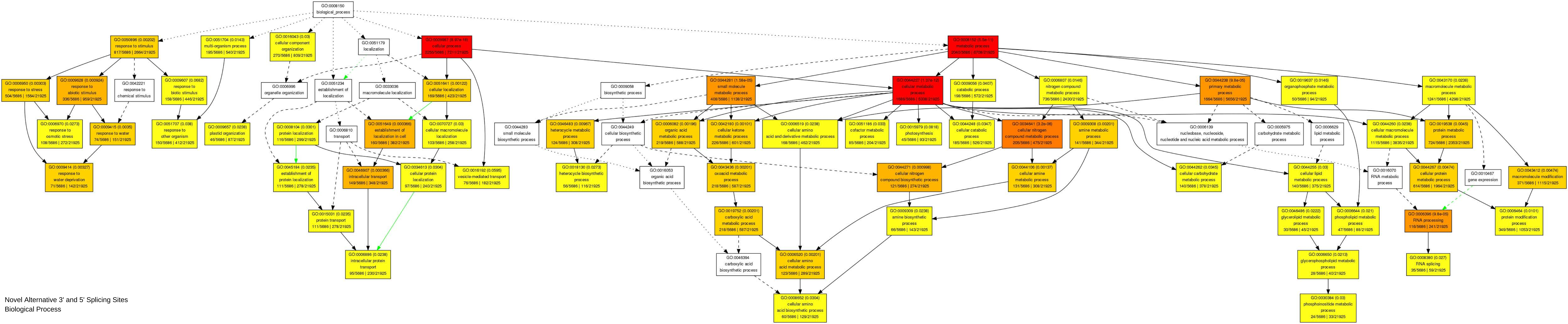

Novel Alternative 3' and 5' Splicing Sites
Biological Process

Novel Alternative 3' and 5' Splicing Sites
Cellular Component

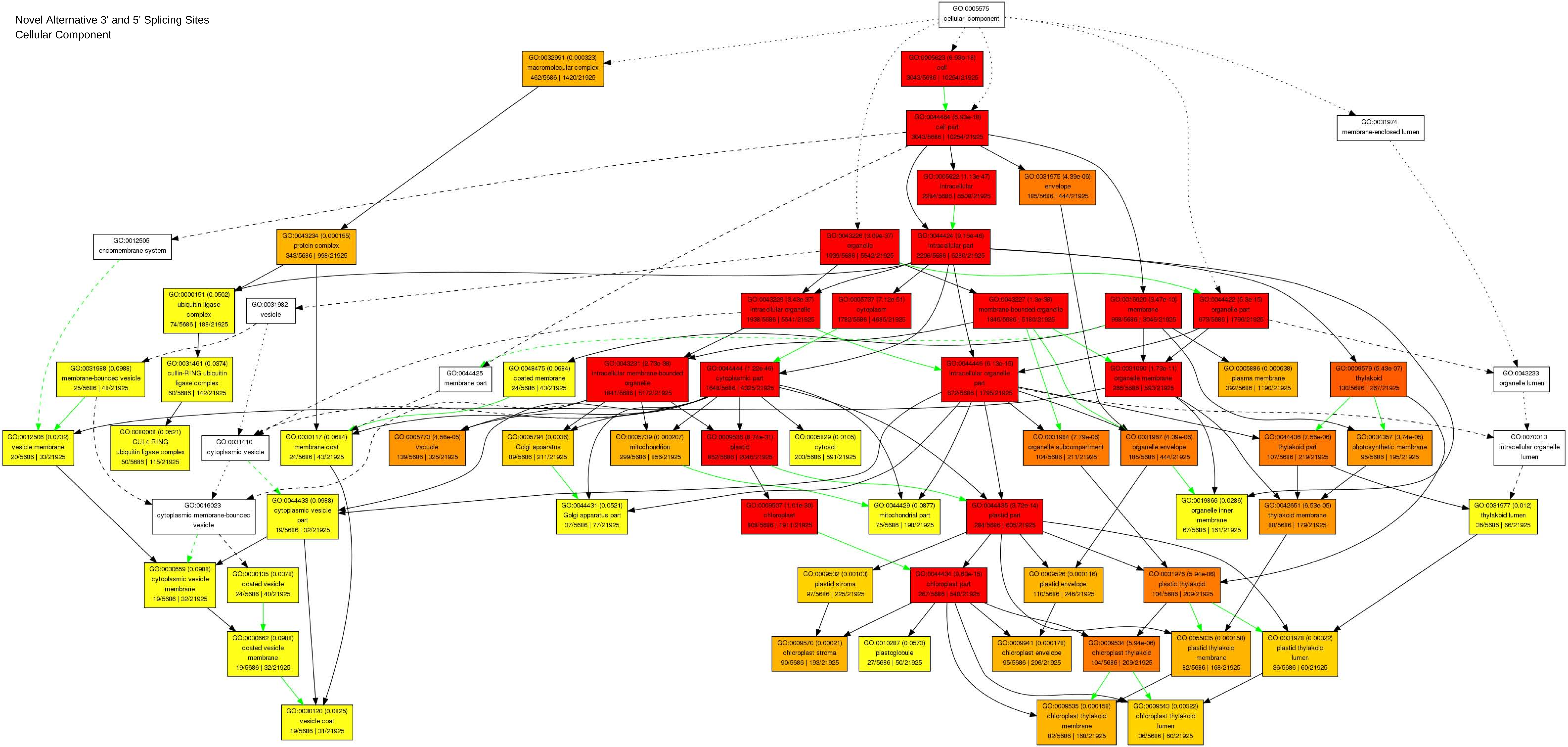

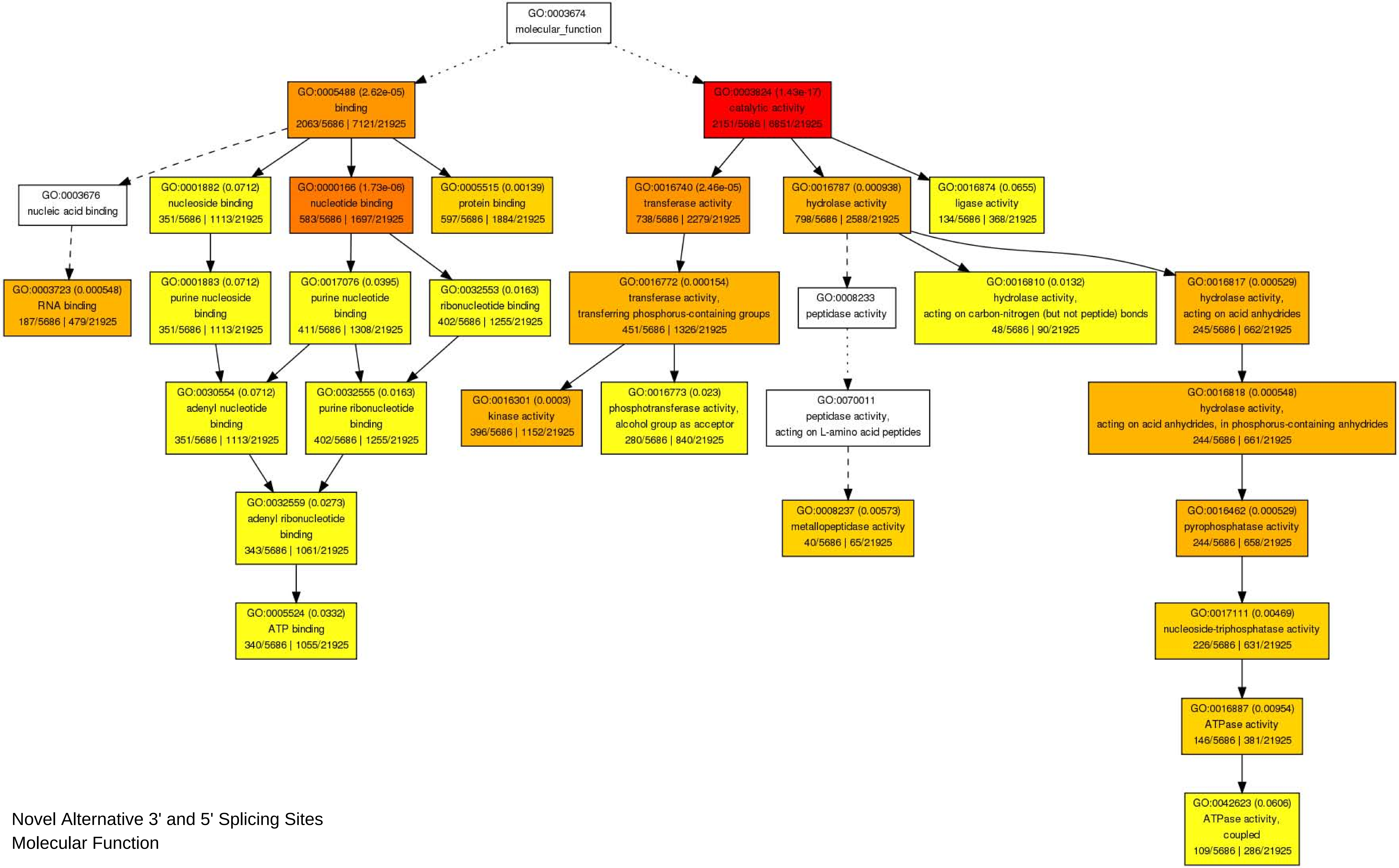

Novel Alternative 3' and 5' Splicing Sites
Molecular Function
